# Machine Learning–Augmented Traditional Analysis of Lactate vs Lactate-to-Albumin Ratio for Predicting Mortality Risk in Patients With Sepsis: Large-Scale Retrospective Study

**DOI:** 10.2196/82230

**Published:** 2026-07-16

**Authors:** Xiaodi Wang, Yi Xu, Weijun Xiao, Peng Dou, Yunxia Huang, Muhan Cao, Liang Luo, Qinghua Hou

**Affiliations:** 1 Department of Neurology, Clinical Neuroscience Center, the 7th Affiliated Hospital, Sun Yat-Sen University Shenzhen, Guangdong China; 2 Department of Rehabilitation medicine, the 7th Affiliated Hospital, Sun Yat-Sen University Shenzhen, Guangdong China; 3 Department of Epidemiology and Statistics, Clinical Medical Research Center, the 7th Affiliated Hospital, Sun Yat-Sen University Shenzhen, Guangdong China; 4 Department of Critical Care Medicine, the 7th Affiliated Hospital, Sun Yat-Sen University Shenzhen, Guangdong China

**Keywords:** biomarker, eICU Collaborative Research Database, eICU database, lactate-to-albumin ratio, LAR, machine learning, mortality prediction, sepsis

## Abstract

**Background:**

Effective risk stratification in sepsis remains a critical clinical challenge. Serum lactate is a cornerstone biomarker of metabolic dysfunction, yet its predictive limitations—particularly in patients without severe hyperlactatemia—are well recognized. The lactate-to-albumin ratio (LAR), a composite mixed-unit index integrating markers of acute metabolic dysfunction and systemic inflammation, has emerged as a promising predictor; however, its incremental discriminative advantage over lactate had not been formally tested in a large multicenter cohort using paired statistical methodology.

**Objective:**

This study aims to determine whether LAR provides statistically significantly higher prediction of 28-day mortality than lactate alone in adult intensive care unit (ICU) patients with sepsis, using threshold effect analysis, restricted cubic splines, DeLong test, and 9 interpretable machine learning models.

**Methods:**

We conducted a retrospective analysis of 3637 adult patients with sepsis from the multicenter eICU Collaborative Research Database (eICU-CRD; 208 hospitals, United States, 2014-2015). The primary outcome was 28-day all-cause in-hospital mortality among patients surviving the initial 48-hour ICU admission period. We used multivariable logistic regression (LR), Cox proportional-hazards regression, threshold effect analysis, restricted cubic spline modeling, DeLong test for area under the receiver operating characteristic curve (AUC) comparison, and machine learning models evaluated with Shapley additive explanations (SHAP) for interpretability. The cohort was divided 70/30 (stratified) into training and held-out test sets; the Synthetic Minority Oversampling Technique was applied exclusively within the training partition to prevent data leakage.

**Results:**

LAR consistently demonstrated stronger and more stable associations with mortality than lactate across all subgroups. DeLong test confirmed statistically significantly higher AUC for LAR: 28-day hospital mortality (AUC_LAR_=0.646, 95% CI 0.623-0.670 vs AUC_lactate_=0.617, 95% CI 0.593-0.641; Z=6.37; *P*<.001; ΔAUC=0.029) and 28-day ICU mortality (AUC_LAR_=0.642 vs AUC_lactate_=0.621; Z=3.71; *P*<.001). A nominally significant Acute Physiology and Chronic Health Evaluation IV (APACHE IV) × LAR interaction (hospital mortality, *P* for interaction=.02) indicated stronger LAR prognostic effects in lower-severity patients (APACHE IV≤70), representing within-biomarker effect modification requiring prospective validation. Among 9 machine learning models for ICU mortality, LR, random forest (RF), and gradient-boosting decision tree (GBDT) achieved the 3 highest AUCs (0.727, 0.726, and 0.725); Light Gradient Boosting Machine (LightGBM) demonstrated the best calibration (Brier score 0.096, the only model below the null Brier of 0.101 at the natural prevalence of 11.4%); GBDT achieved the highest precision-recall AUC (0.293). SHAP identified LAR among the top 10 predictive features in 3 of 4 models for hospital mortality (RF rank 4, LR rank 7, and LightGBM rank 8) and 1 of 4 for ICU mortality (RF rank 4).

**Conclusions:**

LAR demonstrates statistically significantly higher discrimination than lactate alone for 28-day sepsis mortality prediction. LAR may offer greater prognostic utility in patients without severe hyperlactatemia, a population in whom early risk stratification may be particularly relevant.

## Introduction

Lactate is a crucial biomarker reflecting metabolic dysfunction and tissue hypoxia [1]. The lactate-to-albumin ratio (LAR), as a novel composite biomarker, provides a more comprehensive perspective for clinical prognosis by combining lactate—indicating tissue hypoperfusion—with albumin, reflecting inflammatory and nutritional status [2]. Current evidence suggests that both lactate and LAR are closely associated with poor outcomes in various critical illnesses, particularly in patients with acute myocardial infarction, where elevated lactate levels and LAR are significantly correlated with higher 28-day mortality [3]. Furthermore, LAR has been proven to be an effective predictor of in-hospital mortality in patients with acute kidney injury [4], and it also demonstrates significant prognostic value in other patients [5,6].

Sepsis is a life-threatening condition characterized by a dysregulated host response to infection, leading to organ dysfunction [7]. Epidemiological studies have shown that sepsis is a leading cause of death globally, accounting for approximately 19.7% of all deaths worldwide [8]. The global annual incidence is estimated at 49 million cases, with approximately 11 million deaths. Mortality rates vary significantly across regions: US data indicate approximately 2 million sepsis cases annually with 270,000 deaths [9], while German studies report an 11%-point prevalence of sepsis in intensive care units (ICUs), with mortality rates reaching 48.4% in ICUs and 55.2% in hospitals [10]. Overall, sepsis mortality rates range from 20% to 30%, increasing to over 50% in cases of septic shock [11,12]. These regional variations may be attributed to differences in health care accessibility and quality [13].

Although lactate and LAR have shown potential predictive value in sepsis outcomes, their comparative value remains incompletely characterized. Prior studies demonstrated that LAR outperforms lactate alone in predicting hospital mortality (area under the receiver operating characteristic curve [AUC] 0.74 vs 0.70) and showed significant value in predicting 28-day mortality [14]. However, interpreting this association is complex, primarily because elevated lactate in patients with sepsis may result from multiple pathophysiological mechanisms, including excessive glycolysis, tissue hypoperfusion, medication effects (eg, epinephrine), and organ dysfunction [15-17]. Furthermore, the reliability of lactate as an indicator of tissue hypoxia is debatable, showing no significant correlation with central venous oxygen saturation [18]. Critically, although prior studies have described LAR as numerically superior to lactate, none have formally verified this advantage through paired statistical testing—leaving the clinical relevance of this difference unsubstantiated.

To address these gaps, we conducted this study using the Philips eICU Collaborative Research Database (eICU-CRD), a large-scale multicenter ICU database, to rigorously investigate the association between lactate and LAR with 28-day mortality in patients with sepsis. This study uniquely applies the DeLong test for formal paired AUC comparison, threshold effect analysis with bootstrap validation, restricted cubic spline modeling, and 9 interpretable machine learning models with Shapley additive explanations (SHAP) analyses, to comprehensively evaluate and formally compare the prognostic utility of lactate and LAR. This represents the first study to formally test whether LAR’s numerical superiority over lactate achieves statistical significance in a large multicenter sepsis cohort.

## Methods

### Study Design and Data Source

This retrospective observational cohort study used data from the eICU-CRD v2.0, a publicly available multicenter database sourced from the Philips eICU telehealth program [19]. The database contains clinical data from 200,859 patients with critical illness admitted to 208 hospitals across the United States between 2014 and 2015, including demographic characteristics, physiological parameters from bedside monitors, laboratory test results, disease severity scores, and comorbidities. The study design and reporting adhered to the STROBE (Strengthening the Reporting of Observational Studies in Epidemiology) guidelines [20].

### Study Population and Recruitment

We included all adult patients (aged 18 years or older) diagnosed with sepsis on their first ICU admission. Sepsis was identified using the *International Classification of Diseases, Ninth Revision* (*ICD-9*) diagnostic codes (995.91 for sepsis, 995.92 for severe sepsis, and 785.52 for septic shock), combined with infection-related admission diagnoses from the APACHE IV scoring system in the eICU-CRD [21]. We note that this case-identification strategy differs from the strict Sepsis-3 definition [22], as preadmission baseline Sequential Organ Failure Assessment (SOFA) scores are not routinely available in the eICU-CRD; our cohort, therefore, represents patients with critical illness with documented infection and organ dysfunction requiring ICU admission.

**Figure 1:**
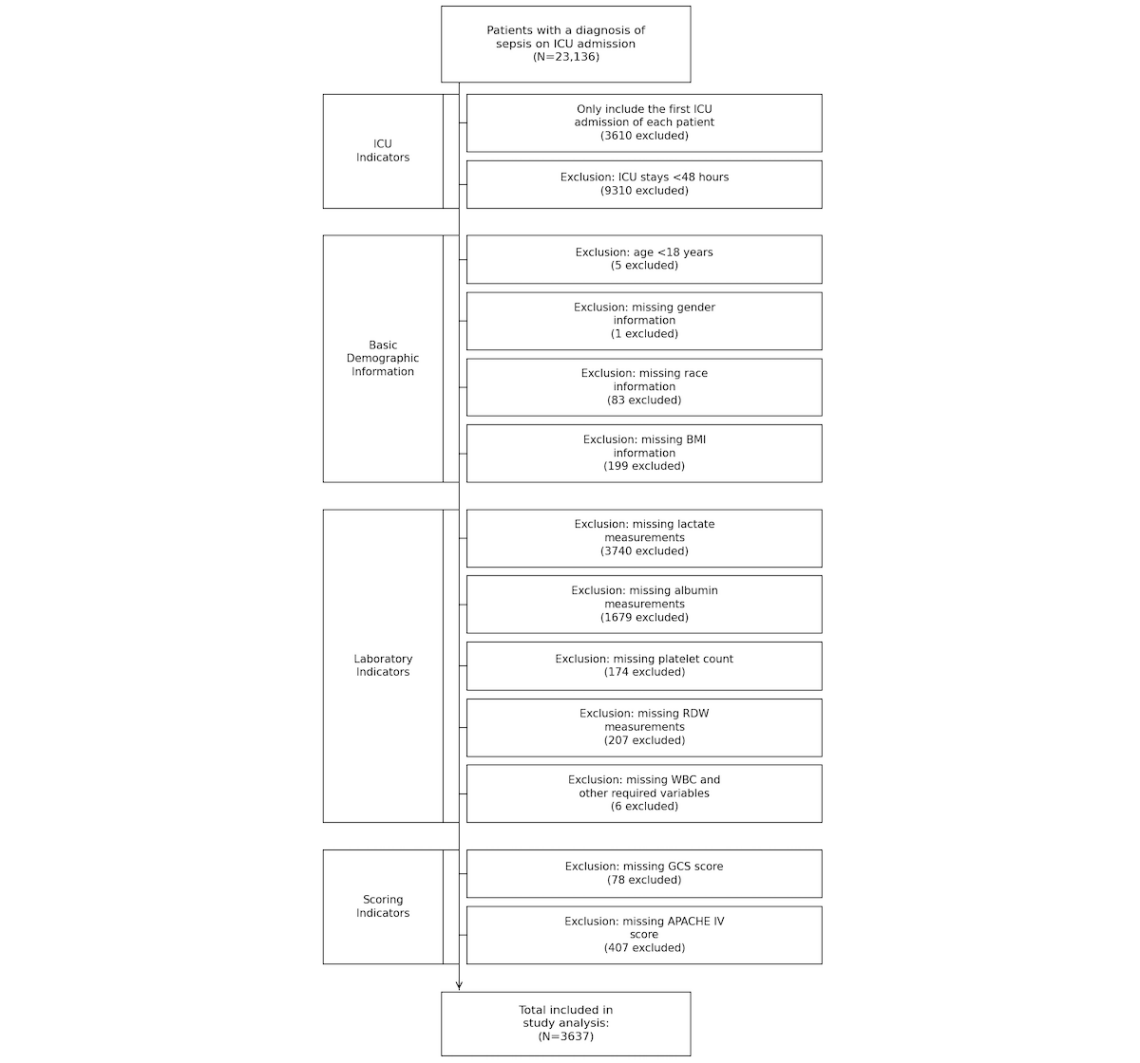
Flow diagram of study population selection from the eICU Collaborative Research Database (208 US hospitals, 2014-2015) for a retrospective cohort study of adult patients with sepsis. Patients were included based on the International Classification of Diseases, Ninth Revision (ICD-9) sepsis codes (995.91, 995.92, and 785.52) plus Acute Physiology and Chronic Health Evaluation IV (APACHE IV) infection-related admission diagnoses, first intensive care unit (ICU) admission only, ICU length of stay ≥48 hours, being age ≥18 years, and complete data for all primary exposures and key covariates. Exclusion counts with percentages relative to the total sepsis screen cohort (N=23,136) are shown at each step. Final analytic cohort: n=3637. GCS: Glasgow Coma Scale; RDW: red cell distribution width; WBC: white blood cell.

Exclusion criteria were (1) nonsepsis primary diagnosis; (2) nonfirst ICU admission; (3) ICU length of stay <48 hours, applied to ensure adequate time for baseline biomarker stabilization and data collection; (4) aged <18 years; (5) undetermined gender, race/ethnicity, or BMI; and (6) missing records for any of the following: serum lactate, albumin, platelet count, red cell distribution width (RDW), Glasgow Coma Scale (GCS) score, or APACHE IV score. Patients with missing white blood cell (WBC) count as the sole missing variable were retained in the analysis via a missing-indicator dummy variable. The patient screening process is shown in Figure 1.

### Data Collection

Comprehensive clinical data were extracted from the eICU-CRD for the period within 24 hours of ICU admission. The following data were collected: (1) demographic characteristics: gender, age, ethnicity, and BMI; (2) physiological indicators: body temperature, respiratory rate, heart rate, and mean arterial pressure (MAP); (3) laboratory test results: exposure variables—serum lactate (mmol/L) and serum albumin (g/dL); covariates—platelet count, RDW, WBC, serum creatinine (mg/dL), and total bilirubin (mg/dL); (4) disease severity scores: GCS score, SOFA score, and APACHE IV score; and (5) comorbidities extracted from the APACHE IV scoring system: AIDS, hepatic failure, lymphoma, metastatic cancer, leukemia, immunosuppression, and diabetes.

LAR was calculated as: LAR = serum lactate (mmol/L) ÷ serum albumin (g/dL). As lactate and albumin use different measurement units, the LAR is a mixed-unit index (mmol/L per g/dL)—not a dimensionless ratio. Standardized unit reporting across all eICU-CRD centers ensures comparability. Biomarker values reflect the first recorded measurement within 24 hours of ICU admission; for emergency department–transferred patients, this measurement may predate ICU admission by 2-4 hours. Hypoalbuminemia was defined as albumin <3.0 g/dL. Lactate was categorized as: low (<2 mmol/L), middle (2 to <4 mmol/L), and high (≥4 mmol/L).

### Outcomes

The primary outcome was 28-day all-cause in-hospital mortality among patients surviving the initial 48-hour ICU admission period, with patients administratively censored at 28 days from ICU admission. Patients alive at 28 days were censored regardless of subsequent hospital discharge status. ICU mortality—defined as death occurring before ICU discharge within 28 days of ICU admission, with the same 28-day administrative censoring—was the secondary outcome. Given the exclusion of patients with ICU stays <48 hours, the modeled prediction window effectively spans days 3 through 28; this temporal constraint should be considered when interpreting model applicability to unselected ICU populations.

### Statistical Analysis

#### Overview

Continuous variables are expressed as mean (SD) or median (IQR); categorical variables as n/N (%). For comparisons across lactate or LAR strata, 1-way ANOVA was used for continuous variables and chi-square tests for categorical data.

#### Multivariable Logistic Regression

Multivariable binary logistic regression (LR) assessed associations between LAR, albumin, lactate, and 28-day binary mortality outcomes (administratively censored at day 28), reporting odds ratios (ORs) with 95% CIs. Both unadjusted (Model 1) and fully adjusted (Model 2) models were reported.

#### Cox Proportional-Hazards Regression

Multivariable Cox regression with 28-day administrative censoring was used for subgroup analyses and threshold effect analyses, reporting hazard ratios (HRs) with 95% CIs. Proportional hazards assumptions were verified using Schoenfeld residuals (all *P*>.05). Covariates for both LR and Cox models included: age, sex, ethnicity, BMI, body temperature, respiratory rate, heart rate, MAP, platelet count, RDW, WBC, GCS score, SOFA score, APACHE IV score, and comorbidities (AIDS, hepatic failure, lymphoma, metastatic cancer, leukemia, immunosuppression, and diabetes).

#### Threshold Effect Analysis

Piecewise Cox regression identified threshold break points by maximum log-likelihood, comparing single-segment vs 2-segment models using log-likelihood ratio tests. Bootstrap resampling (500 iterations, same fully adjusted model specification as the primary analysis) estimated 95% CIs for threshold points.

#### Formal AUC Comparison

DeLong test for correlated receiver operating characteristic (ROC) curves (*pROC* package, R version 4.3.0; R Foundation for Statistical Computing) formally compared LAR vs lactate as stand-alone predictors; *P*<.05 was considered statistically significant. This addresses the requirement for formal paired statistical testing to substantiate claims of superior discrimination.

#### Subgroup Interactions

*P* values for biomarker × subgroup interaction were calculated by including a multiplicative interaction term in Cox models.

#### Additional Analyses

**Figure 2:**
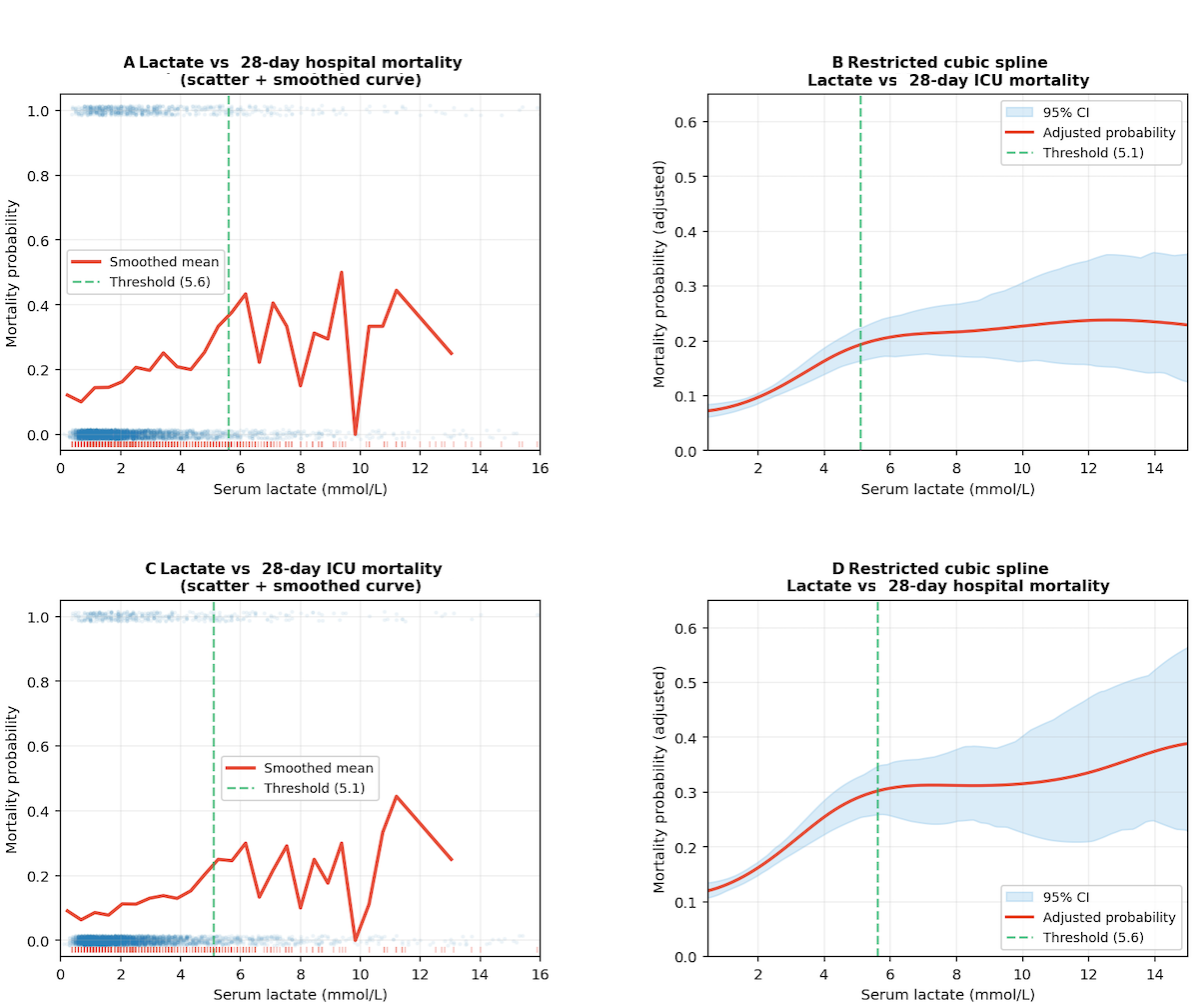
Dose-response relationship between serum lactate levels and 28-day mortality in adult patients with sepsis (retrospective cohort study, eICU Collaborative Research Database, n=3637; United States, 2014-2015). (A) Scatter plot with locally estimated scatterplot smoothing (LOESS)—lactate vs 28-day hospital mortality. (B) Restricted cubic spline—28-day intensive care unit (ICU) mortality; 95% CI (dotted lines). (C) Restricted cubic spline—28-day hospital mortality. (D) Scatter plot—28-day ICU mortality. All splines adjusted for age, sex, ethnicity, BMI, vital signs, severity scores, and comorbidities. Rug plots indicate lactate measurement distribution. Vertical dashed lines indicate identified threshold break points.

**Figure 3:**
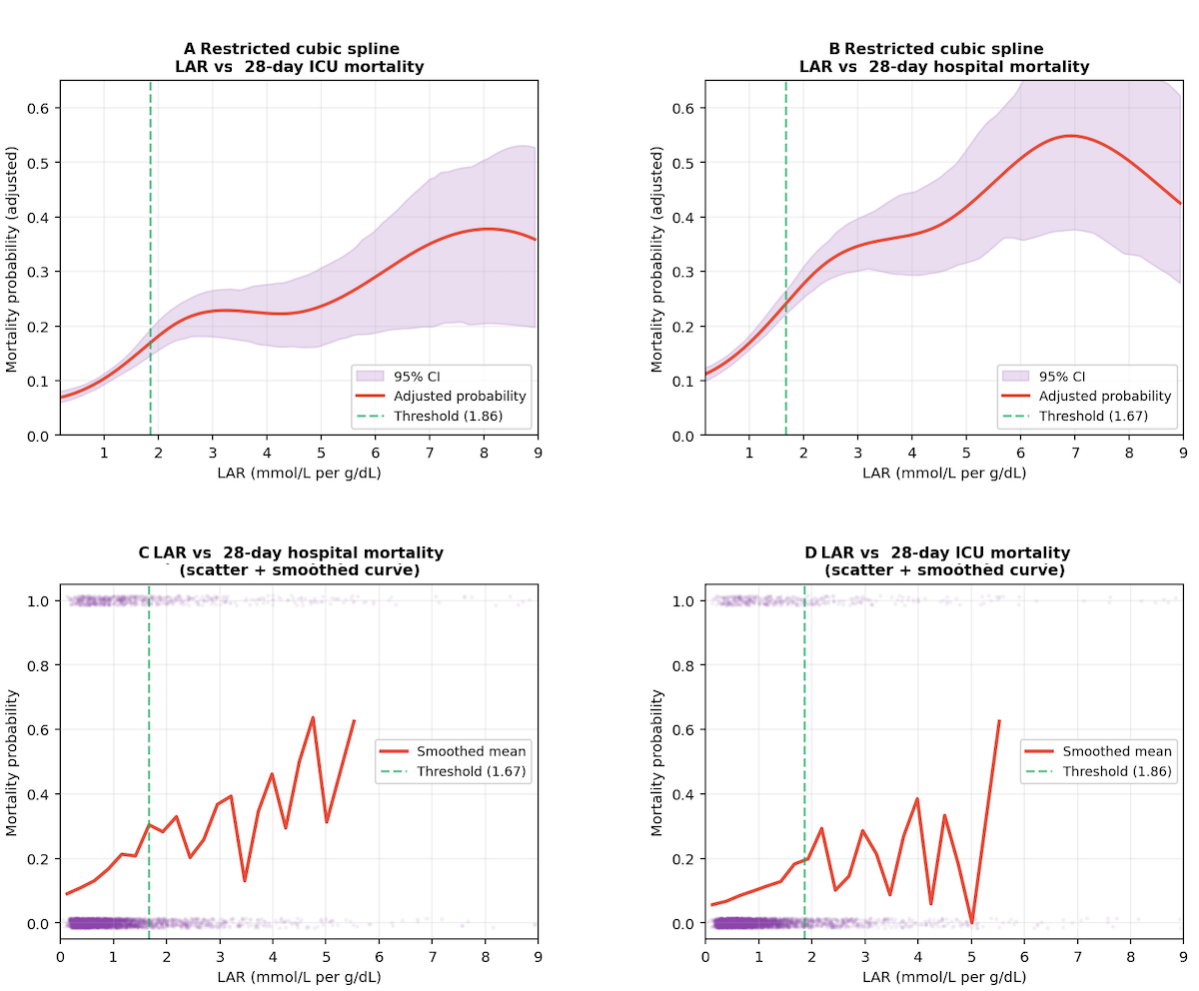
Nonlinear association between the lactate-to-albumin ratio (LAR, mmol/L per g/dL) and 28-day mortality in adult patients with sepsis (retrospective cohort study, eICU Collaborative Research Database, n=3637; United States, 2014-2015). (A and B) Restricted cubic splines for intensive care unit (ICU) and hospital mortality with 95% CI. (C and D) Scatter plots. LAR shows a sustained risk gradient across 0-5 units, contrasting with lactate’s plateau. Vertical dashed lines indicate threshold breakpoints. All analyses adjusted as in Figure 2.

Variance inflation factors (VIF) assessed multicollinearity. E-values quantified robustness to unmeasured confounding. Decision curve analysis (DCA) evaluated net clinical benefit. Restricted cubic splines (5 knots) modeled nonlinear dose-response relationships (Figures 2 and 3), adjusted for all covariates listed above. A 2-sided α level of .05 was used for all tests. Analyses were performed in EmpowerStats and R (version 4.3.0).

#### Missing Data

For traditional statistical analyses, patients missing primary exposures or key covariates were excluded from the cohort per Figure 1. For remaining covariates with >1% missingness—body temperature (2.6% missing) and WBC (8.4% missing)—missing indicator variables were introduced to preserve sample size. For machine learning models, k-nearest neighbors (KNN) imputation (k=5) was applied to continuous features and mode imputation to categorical features within the training partition only (see Machine Learning Modeling section).

#### Sensitivity Analysis for Measurement-Timing Misclassification

A sensitivity analysis was conducted, excluding 142 patients (3.9% of the cohort) whose first biomarker measurement was recorded more than 6 hours before ICU admission. Classical preadmission immortal-time bias is largely mitigated by the cohort’s inclusion criterion requiring ICU length of stay ≥48 hours; this additional analysis therefore specifically probes whether biomarker values obtained substantially before ICU admission—and thus potentially unrepresentative of ICU admission physiology—introduce measurement-timing misclassification into the exposure definition. This sensitivity analysis complements the 48-hour exclusion criterion by addressing a distinct bias mechanism. The exclusion produced no material change in discriminative performance (ΔAUC<0.01 for both outcomes), confirming that differential measurement timing does not substantively affect the results.

### Machine Learning Modeling

The cohort (N=3637) was divided into a training set (n=2545, 70%) and a shared held-out test set (n=1092, 30%) using a single stratified random split (seed 42, stratified on 28-day ICU mortality) to preserve class balance; as stratification was performed on ICU rather than hospital mortality, the test-set hospital mortality prevalence (197/1092, 18%) differs marginally from the overall cohort prevalence (666/3637, 18.3%). The same held-out test set was used for the evaluation of both ICU and hospital mortality models. KNN imputation (k=5) and standard scaling of continuous features were fitted exclusively on the training set and applied to the held-out test set without refitting, preventing data leakage. Synthetic Minority Oversampling Technique (SMOTE) oversampling was applied exclusively within the training partition and was not applied to the held-out test set in any form; all performance metrics were evaluated on the natural, unbalanced held-out test set (n=1092). Binary categorical variables (diabetes, hepatic failure, immunosuppression, lymphoma, metastatic cancer, leukemia, AIDS, and sex) were excluded from standard scaling to ensure correct discrete representation in SHAP analyses. For LR, feature importance was assessed using the mean absolute SHA*P* value. SHA*P* values were computed using the same preprocessed feature representations as used for model training. As SHA*P* values reflect each feature’s marginal contribution to the model output—independently of the absolute measurement scale of the input features—this approach allows direct comparison of feature importance across both standardized continuous and binary variable types without requiring additional normalization. The same metric—mean absolute SHA*P* value—was applied consistently to models to ensure comparability of feature importance rankings. Noninterpretable V-code metadata fields were excluded (ΔAUC≤0.005).

A total of 9 supervised classifiers were trained: random forest (RF), extreme gradient boosting (XGBoost), LightGBM, GBDT, LR, support vector machine (SVM), KNN, decision tree, and adaptive boosting (AdaBoost). Hyperparameters were tuned using 5-fold cross-validation within the training set only. For the machine learning LR model specifically, L2 (ridge) regularization was applied; the inverse regularization strength (C) was selected via grid search over (0.001, 0.01, 0.1, 1, 10, 100) using 5-fold cross-validation on the training set. All 3 correlated features (LAR, lactate, and albumin) were retained in the machine learning feature set; the L2 penalty jointly shrinks collinear coefficient estimates and yields stable predictions despite their algebraic dependence, which is distinct from the collinearity problem in unpenalized multivariable LR, where simultaneous inclusion of all 3 variables would preclude valid coefficient identification. All performance metrics reported (AUC, precision-recall AUC (PR-AUC), Brier score, positive-class F1-score, sensitivity, and specificity) reflect evaluation on the held-out test set exclusively. SHAP bar plots display the top 10 individual features with no aggregated residual bar, and binary categorical variables are rendered with 2 discrete color values (high or low) rather than a continuous color gradient.

For external validation, adult patients with sepsis were extracted from the Medical Information Mart for Intensive Care IV (MIMIC-IV; v2.2) applying the same inclusion and exclusion criteria as the eICU-CRD cohort, including the requirement for ICU length of stay ≥48 hours. Sepsis was identified using *ICD-9* codes (995.91, 995.92, and 785.52) and *ICD-10* codes (A40.x and A41.x), supplemented by infection-related admission diagnoses. In MIMIC-IV, where the structured APACHE IV admission-diagnosis field available in eICU-CRD is not present, infection-related admission diagnoses were ascertained exclusively through ICD billing codes in the diagnoses_icd table, without supplementary structured admission-diagnosis classification. The 18 clinical features shared between the 2 databases were extracted and preprocessed identically to the eICU-CRD cohort. Continuous variables were scaled using the StandardScaler fitted exclusively on the eICU-CRD training partition and applied directly to MIMIC-IV data without refitting, preventing information leakage from the validation set. Of note, APACHE IV score—the dominant predictor in all eICU-trained models—is not a native variable in MIMIC-IV and was not among the 18 shared features.

The 18 shared clinical features comprised vital signs (heart rate, respiratory rate, MAP, and temperature), laboratory values (lactate, albumin, LAR, WBC, creatinine, blood urea nitrogen, hematocrit, and bilirubin), organ dysfunction scores (SOFA and GCS), and clinical indicators (vasopressor use, urine output, platelet count, and RDW). In MIMIC-IV, APACHE IV was structurally absent for all patients. When applied to a wholly absent column, the KNNImputer fitted on the eICU-CRD training partition computed interpatient distances using the 17 remaining shared features and imputed each patient’s APACHE IV as the mean of its k=5 nearest neighbors’ values from the training set. Because the MIMIC-IV sepsis cohort occupies a broadly similar region of the 17-feature covariate space as the eICU training patients, the resulting nearest-neighbor APACHE IV means were tightly clustered around the conditional training-set mean, effectively assigning a near-constant severity estimate across all MIMIC-IV patients and substantially suppressing the discriminative contribution of APACHE IV in the external validation setting. The discriminative contribution of APACHE IV in the external validation setting was therefore substantially attenuated, and model discrimination relied predominantly on the remaining shared clinical features. Models were deployed without recalibration.

### Ethical Considerations

This study used data from the eICU-CRD, which was approved by the PhysioNet Review Board (data use agreement record number 66014966). The database complies with HIPAA (Health Insurance Portability and Accountability Act) Safe Harbor provisions. As this was a retrospective analysis of a deidentified publicly available database, individual informed consent was waived; local ethics committee approval was also waived in accordance with institutional guidelines. The researcher (YX) completed the required data access training certification (certificate number 66014966). The use of MIMIC-IV for external validation was approved by the Institutional Review Boards of Beth Israel Deaconess Medical Center (protocol number 2001-P-001699/14) and the Massachusetts Institute of Technology (number 0403000206). The requirement for individual informed consent was waived due to the retrospective nature of the analysis. Patient data were deidentified prior to access. All study procedures were conducted in accordance with the Declaration of Helsinki.

## Results

### Characteristics of the Study Population

Among the 3637 critically ill patients with sepsis included, 28-day hospital mortality was 18.3% (666/3637) and 28-day ICU mortality was 11.4% (415/3637). Elevated lactate was significantly associated with higher APACHE IV scores (mean 73.84, SD 23.82 to mean 96.44, SD 29.13; *P*<.001), higher respiratory rate, and higher heart rate (all *P*<.001). LAR demonstrated broader prognostic utility, additionally reflecting hemodynamic instability through blood pressure alterations (*P*=.05) and nutritional status through BMI correlation (*P*=.008). Notably, 28-day hospital mortality increased more steeply across LAR tertiles—from 11.2% to 28.5% (*P*<.001)—compared with 13.5% to 29.2% across lactate groups (*P*<.001), suggesting broader prognostic separation. Detailed baseline characteristics are presented in Table 1.

**Table 1:** Baseline characteristics of the study population. *P* values were calculated using ANOVA for continuous variables and chi-square tests for categorical variables. LAR^a^ tertiles: T1≤0.59, T2=0.59-1.14, T3>1.14.

Variable			Lactate groups					*P* value			LAR groups						*P* value	
			Low (<2 mmol/L) (n=1860)	Middle (≥2 to <4 mmol/L) (n=1167)		High (≥4 mmol/L) (n=610)					Low (T1) (n=1212)			Middle (T2) (n=1213)		High (T3) (n=1212)		
Demographics																		
	Age (years), mean (SD)	64.65 (15.13)		66.45 (15.27)	64.48 (15.8)		.003			64.22 (15.42)			66.31 (15.03)		65.06 (15.41)		.003	
	Sex (male), n (%)	936 (50.3%)		608 (52.1%)	309 (50.7%)		.61			589 (48.6%)			645 (53.2%)		618 (51.0%)		.08	
	BMI (kg/m^2^), mean (SD)	29.31 (8.83)		29.19 (9.56)	28.41 (8.87)		.10			29.68 (8.81)			29.15 (9.02)		28.53 (9.37)		.008	
Ethnicity, n (%)									.03									.007
	White	1501 (80.7%)		928 (79.5%)	462 (75.7%)					976 (80.5%)			998 (82.3%)		918 (75.7%)			
	African American	167 (9.0%)		111 (9.5%)	65 (10.7%)					116 (9.6%)			90 (7.4%)		137 (11.3%)			
	Hispanic	102 (5.5%)		64 (5.5%)	37 (6.1%)					70 (5.8%)			62 (5.1%)		72 (5.9%)			
	Asian	45 (2.4%)		30 (2.6%)	27 (4.4%)					27 (2.2%)			32 (2.6%)		44 (3.6%)			
Vital signs, mean (SD)																		
	Heart rate (beats/min)	112.07 (28.33)		118.1 (27.86)	123.92 (27.16)		<.001			110.22 (28.43)			115.69 (28.74)		122.03 (26.56)		<.001	
	Respiratory rate (breaths/min)	29.49 (14.86)		31.31 (13.86)	32.43 (13.95)		<.001			28.93 (15.01)			30.74 (14.39)		32.01 (13.73)		<.001	
	Mean blood pressure (mm Hg)	77.52 (44.65)		76.28 (44.52)	76.18 (48.05)		.70			79.27 (45.06)			76.69 (44.85)		74.74 (45.58)		.05	
Laboratory values, mean (SD)																		
	Platelets (×10^9^/L)	208.44 (112.9)		193.47 (114.11)	169.79 (119.51)		<.001			210.59 (108.37)			201.16 (111.17)		179.81 (123.49)		<.001	
	RDW^b^ (%)	16.03 (2.56)		16.09 (2.66)	16.6 (2.9)		<.001			15.87 (2.48)			16.02 (2.56)		16.54 (2.87)		<.001	
	WBC^c^ (×10^9^/L)	15.66 (11.7)		16.95 (10.89)	17.2 (13.85)		.002			14.64 (9.42)			16.91 (12.74)		17.44 (12.90)		<.001	
	Lactate (mmol/L)	1.23 (0.41)		2.74 (0.56)	6.75 (2.95)		<.001			—^d^			—		—		—	
	LAR (mmol/L per g/dL)	0.54 (0.25)		1.2 (0.45)	3.06 (1.76)		<.001			0.39 (0.12)			0.82 (0.16)		2.29 (1.49)		<.001	
Disease severity scores																		
	APACHE IV^e^ score, mean (SD)	73.84 (23.82)		81.68 (25.75)	96.44 (29.13)		<.001			70.84 (23.00)			77.72 (24.44)		91.81 (27.87)		<.001	
	SOFA^f^ score, mean (SD)	4.93 (2.89)		5.54 (2.96)	7.36 (3.4)		<.001			5.00 (3.00)			6.30 (3.30)		8.50 (3.90)		<.001	
	GCS^g^ score, median (IQR)	14 (10-15)		14 (9-15)	12 (8-15)		<.001			14 (11-15)			14 (10-15)		12 (7-15)		<.001	
Comorbidities, n (%)																		
	Diabetes	426 (22.9%)		308 (26.4%)	164 (26.9%)		.04			287 (23.7%)			303 (25.0%)		308 (25.4%)		.59	
	Hepatic failure	35 (1.9%)		26 (2.2%)	35 (5.7%)		<.001			15 (1.2%)			30 (2.5%)		53 (4.4%)		<.001	
	Immunosuppression	141 (7.6%)		107 (9.2%)	62 (10.2%)		.09			90 (7.4%)			103 (8.5%)		124 (10.2%)		.06	
Outcomes, n (%)																		
	28-day ICU^h^ mortality	153 (8.2%)		139 (11.9%)	125 (20.5%)		<.001			81 (6.7%)			112 (9.2%)		223 (18.4%)		<.001	
	28-day hospital mortality	251 (13.5%)		237 (20.3%)	178 (29.2%)		<.001			136 (11.2%)			184 (15.2%)		345 (28.5%)		<.001	

^a^LAR: lactate-to-albumin ratio.

^b^RDW: red cell distribution width.

^c^WBC: white blood cell.

^d^Not applicable/Not available.

^e^APACHE IV: Acute Physiology and Chronic Health Evaluation IV.

^f^SOFA: Sequential Organ Failure Assessment.

^g^GCS: Glasgow Coma Scale.

^h^ICE: intensive care unit.

### Subgroup Analysis

Cox proportional-hazards regression revealed that LAR consistently demonstrated higher HRs than lactate across all clinical subgroups examined (Table 2). Among patients with fever (temperature ≥37.2 °C), LAR showed the highest predictive value for 28-day ICU mortality (HR 1.71, 95% CI 1.46-2.01; *P*<.001), compared to lactate (HR 1.29, 95% CI 1.19-1.39; *P*<.001). For patients with liver disease, LAR predicted 28-day ICU mortality (HR 1.91, 95% CI 1.29-2.82; *P*=.001) substantially more strongly than lactate (HR 1.28, 95% CI 1.10-1.49; *P*=.002).

Formal interaction testing identified a nominal APACHE IV × LAR interaction for 28-day hospital mortality (*P* for interaction=.02). The nominally significant interaction term indicates that the prognostic effect of LAR was significantly stronger in patients with lower APACHE IV scores (≤70; HR 1.77, 95% CI 1.42-2.20; *P*<.001) than in higher-severity patients—a finding of within-biomarker effect modification that requires prospective confirmation. For reference, lactate HR in the same subgroup was 1.17 (95% CI 1.05-1.31; *P*=.005); however, this comparison across biomarkers is descriptive only and does not constitute a formal test of between-biomarker superiority. No other subgroup interactions were statistically significant (all *P* for interaction>.05), confirming the broad consistency of LAR’s prognostic utility. Complete subgroup results, including all *P* values for interaction, are presented in Table 2.

**Table 2:** Subgroup analysis of lactate and LAR^a^ for 28-day mortality. P for interaction is shown in the first row of each subgroup and applies to all levels within that subgroup. Results are from multivariable Cox proportional-hazards regression with 28-day administrative censoring, adjusted for age, sex, ethnicity, BMI, body temperature, respiratory rate, heart rate, mean arterial pressure, platelet count, red cell distribution width, white blood cell count, GCS^b^, SOFA^c^, APACHE IV^d^, and comorbidities.

Subgroup		Lactate: 28-day ICU^e^ mortality, HR^f^ (95% CI)	*P* value	Lactate: 28-day hospital mortality, HR (95% CI)	*P* value	LAR: 28-day ICU mortality, HR (95% CI)	*P* value	LAR: 28-day hospital mortality, HR (95% CI)	*P* value	*P* for interaction (LAR × subgroup hospital)		*P* for interaction (LAR × subgroup ICU)	
Sex											.42		.63
	Male	1.13 (1.07-1.18)	<.001	1.12 (1.07-1.17)	<.001	1.37 (1.24-1.51)	<.001	1.41 (1.28-1.55)	<.001				
	Female	1.18 (1.13-1.24)	<.001	1.18 (1.13-1.24)	<.001	1.35 (1.24-1.48)	<.001	1.43 (1.31-1.56)	<.001				
Age (years)											.48		.63
	≤64	1.18 (1.12-1.25)	<.001	1.18 (1.13-1.25)	<.001	1.39 (1.25-1.55)	<.001	1.48 (1.33-1.65)	<.001				
	65-74	1.13 (1.07-1.20)	<.001	1.14 (1.08-1.21)	<.001	1.34 (1.19-1.52)	<.001	1.44 (1.27-1.62)	<.001				
	≥75	1.16 (1.09-1.24)	<.001	1.14 (1.08-1.21)	<.001	1.37 (1.22-1.55)	<.001	1.38 (1.23-1.55)	<.001				
Temperature (°C)											.26		.38
	≤36.4	1.12 (1.07-1.17)	<.001	1.12 (1.07-1.16)	<.001	1.26 (1.15-1.37)	<.001	1.30 (1.20-1.42)	<.001				
	36.5-37.1	1.09 (1.00-1.19)	.04	1.12 (1.04-1.20)	.002	1.28 (1.10-1.48)	.001	1.33 (1.16-1.52)	<.001				
	≥37.2	1.29 (1.19-1.39)	<.001	1.26 (1.17-1.35)	<.001	1.71 (1.46-2.01)	<.001	1.73 (1.49-2.00)	<.001				
GCS score											.78		.59
	3-8	1.12 (1.04-1.21)	.004	1.11 (1.03-1.19)	.006	1.30 (1.12-1.51)	<.001	1.32 (1.15-1.52)	<.001				
	9-12	1.14 (1.05-1.23)	.001	1.12 (1.04-1.20)	.002	1.33 (1.15-1.54)	<.001	1.35 (1.18-1.55)	<.001				
	13-15	1.17 (1.11-1.23)	<.001	1.16 (1.11-1.22)	<.001	1.39 (1.26-1.54)	<.001	1.45 (1.32-1.60)	<.001				
SOFA score											.51		.74
	0-4	1.13 (1.06-1.21)	<.001	1.15 (1.09-1.22)	<.001	1.32 (1.15-1.52)	<.001	1.45 (1.27-1.65)	<.001				
	5-9	1.14 (1.08-1.21)	<.001	1.13 (1.08-1.19)	<.001	1.35 (1.22-1.50)	<.001	1.38 (1.25-1.52)	<.001				
	≥10	1.19 (1.08-1.31)	<.001	1.16 (1.06-1.26)	.001	1.46 (1.22-1.75)	<.001	1.47 (1.24-1.74)	<.001				
APACHE IV tertile											.02^g^		.18
	Low (≤70)	1.14 (0.98-1.32)	.08	1.17 (1.05-1.31)	.005	1.42 (1.07-1.88)	.02	1.77 (1.42-2.20)	<.001				
	Middle (71-85)	1.10 (1.03-1.18)	.006	1.08 (1.01-1.15)	.02	1.26 (1.10-1.45)	.001	1.22 (1.07-1.38)	.002				
	High (≥86)	1.10 (1.06-1.15)	<.001	1.10 (1.06-1.14)	<.001	1.22 (1.13-1.33)	<.001	1.26 (1.16-1.36)	<.001				
Liver disease											.14		.13
	No	1.14 (1.10-1.18)	<.001	1.14 (1.11-1.18)	<.001	1.33 (1.24-1.42)	<.001	1.39 (1.30-1.49)	<.001				
	Yes	1.28 (1.10-1.49)	.002	1.28 (1.10-1.50)	.002	1.91 (1.29-2.82)	.001	1.96 (1.32-2.91)	<.001				
Immunosuppression											.77		.65
	No	1.15 (1.11-1.19)	<.001	1.15 (1.12-1.19)	<.001	1.39 (1.29-1.51)	<.001	1.45 (1.34-1.56)	<.001				
	Yes	1.18 (1.02-1.37)	.02	1.14 (1.00-1.30)	.05	1.26 (1.10-1.45)	<.001	1.36 (1.20-1.54)	<.001				

^a^LAR: lactate-to-albumin ratio.

^b^GCS: Glasgow Coma Scale.

^c^SOFA: Sequential Organ Failure Assessment.

^d^APACHE IV: Acute Physiology and Chronic Health Evaluation IV.

^e^ICU: intensive care unit.

^f^HR: hazards ratio.

^g^Nominally statistically significant interaction (*P*=.02): the prognostic effect of LAR was significantly stronger in the low APACHE IV subgroup (score≤70) than in higher-severity patients, representing within-biomarker effect modification.

### Multivariate Regression Analysis

Lactate-stratified LR analysis (Table 3) revealed that LAR and albumin were consistently associated with 28-day mortality across all subgroups, with the most pronounced effects observed in patients with lactate <2 mmol/L. In this low-lactate subgroup, LAR demonstrated the highest predictive value (28-day ICU mortality: OR 2.89, 95% CI 1.58-5.30; *P*<.001 and 28-day hospital mortality: OR 4.29, 95% CI 2.60-7.06; *P*<.001), while albumin showed a pronounced protective effect (28-day ICU mortality: OR 0.57, 95% CI 0.42-0.76; *P*<.001). Notably, lactate itself was not independently associated with mortality in this subgroup after full adjustment (*P*>.05). For the intermediate lactate group (2 to <4 mmol/L), the effect of LAR remained significant but attenuated (28-day hospital mortality: OR 2.56, 95% CI 1.86-3.51; *P*<.001). In the high-lactate group (≥4 mmol/L), LAR and albumin remained independently associated with 28-day hospital mortality after full adjustment (LAR: OR 1.18, 95% CI 1.07-1.31; *P*=.002). Lactate did not reach statistical significance after adjustment for all covariates in this subgroup (OR 1.04, 95% CI 0.97-1.11; *P*=.26), suggesting that its prognostic contribution in the high-lactate range is largely captured by the composite LAR and other severity markers. These findings confirm that LAR and hypoalbuminemia serve as robust prognostic indicators across the full lactate severity spectrum, whereas lactate’s independent predictive utility is most evident at higher concentrations.

**Table 3:** Multivariate regression analysis of lactate and LAR^a^ for predicting 28-day ICU^b^ and hospital mortality. LAR (mmol/L per g/dL), lactate, and albumin were entered as exposures in 3 separate multivariable logistic regression models within each lactate stratum, each including only 1 exposure variable alongside the same covariate adjustment set, to avoid mathematical collinearity arising from the algebraic dependence of LAR on lactate and albumin. Model 1: unadjusted. Model 2: adjusted for age, sex, ethnicity, BMI, body temperature, respiratory rate, heart rate, mean arterial pressure, platelet count, red cell distribution width, white blood cell count, Glasgow Coma Scale score, Sequential Organ Failure Assessment score, Acute Physiology and Chronic Health Evaluation IV score, and comorbidities (AIDS, hepatic failure, lymphoma, metastatic cancer, leukemia, immunosuppression, and diabetes).

Exposure				Model 1 (unadjusted), OR^c^ (95% CI)		*P* value		Model 2 (adjusted), OR (95% CI)		*P* value
28-day ICU mortality										
	Lactate group (<2 mmol/L)									
		LAR	2.85 (1.59-5.12)		<.001		2.89 (1.58-5.30)		<.001	
		Albumin (g/dL)	0.60 (0.45-0.79)		<.001		0.57 (0.42-0.76)		<.001	
		Lactate (mmol/L)	1.42 (0.94-2.14)		.10		1.31 (0.86-1.99)		.20	
	Lactate group (2 to <4 mmol/L)									
		LAR	1.93 (1.37-2.72)		<.001		2.00 (1.41-2.86)		<.001	
		Albumin (g/dL)	0.60 (0.44-0.81)		<.001		0.57 (0.42-0.78)		<.001	
		Lactate (mmol/L)	1.36 (1.00-1.86)		.05		1.33 (0.97-1.82)		.08	
	Lactate group (≥4 mmol/L)									
		LAR	1.18 (1.07-1.30)		.001		1.18 (1.06-1.30)		.002	
		Albumin (g/dL)	0.69 (0.50-0.95)		.02		0.72 (0.52-1.00)		.05	
		Lactate (mmol/L)	1.07 (1.01-1.14)		.03		1.07 (1.00-1.14)		.04	
	Total (all patients)									
		LAR	1.25 (1.13-1.38)		<.001		1.26 (1.14-1.39)		<.001	
		Albumin (g/dL)	0.62 (0.52-0.74)		<.001		0.61 (0.51-0.73)		<.001	
		Lactate (mmol/L)	1.09 (1.02-1.15)		.005		1.09 (1.03-1.16)		.005	
28-day hospital mortality										
	Lactate group (<2 mmol/L)									
		LAR	4.00 (2.48-6.46)		<.001		4.29 (2.60-7.06)		<.001	
		Albumin (g/dL)	0.54 (0.43-0.68)		<.001		0.50 (0.39-0.63)		<.001	
		Lactate (mmol/L)	1.47 (1.06-2.05)		.02		1.33 (0.95-1.86)		.10	
	Lactate group (2 to <4 mmol/L)									
		LAR	2.38 (1.76-3.22)		<.001		2.56 (1.86-3.51)		<.001	
		Albumin (g/dL)	0.51 (0.40-0.65)		<.001		0.48 (0.37-0.62)		<.001	
		Lactate (mmol/L)	1.39 (1.08-1.79)		.01		1.35 (1.04-1.75)		.02	
	Lactate group (≥4 mmol/L)									
		LAR	1.25 (1.13-1.38)		<.001		1.18 (1.07-1.31)		.002	
		Albumin (g/dL)	0.50 (0.38-0.68)		<.001		0.51 (0.38-0.69)		<.001	
		Lactate (mmol/L)	1.08 (1.02-1.14)		.008		1.04 (0.97-1.11)		.26	
	Total (all patients)									
		LAR	1.40 (1.26-1.54)		<.001		1.43 (1.29-1.59)		<.001	
		Albumin (g/dL)	0.52 (0.45-0.60)		<.001		0.50 (0.43-0.58)		<.001	
		Lactate (mmol/L)	1.10 (1.04-1.16)		.001		1.11 (1.05-1.17)		<.001	

^a^LAR: lactate-to-albumin ratio.

^b^ICU: intensive care unit.

^c^OR: odds ratio.

### Stand-Alone Predictive Performance and Formal AUC Comparison

As stand-alone predictors of 28-day hospital mortality, APACHE IV score demonstrated the highest discrimination (AUC 0.683, 95% CI 0.661-0.705), followed by LAR (AUC 0.646, 95% CI 0.623-0.670), SOFA score (AUC 0.622, 95% CI 0.598-0.645), lactate (AUC 0.617, 95% CI 0.593-0.641), and GCS score (AUC 0.564, 95% CI 0.540-0.587). Consistent ordering was observed for ICU mortality.

DeLong test for correlated ROC curves formally confirmed that LAR has statistically significantly higher discriminative performance than lactate for both outcomes: 28-day hospital mortality (AUC_LAR_=0.646 vs AUC_lactate_=0.617; Z=6.37; *P*<.001; ΔAUC=0.029) and 28-day ICU mortality (AUC_LAR_=0.642 vs AUC_lactate_=0.621; Z=3.71; *P*<.001; ΔAUC=0.021). Although the absolute AUC differences are modest—as expected given that LAR is algebraically derived from lactate—their statistical robustness across both outcome definitions in 3637 patients provides formal evidence that LAR captures prognostic information beyond lactate alone, likely reflecting the independent contribution of hypoalbuminemia as a marker of systemic inflammation, capillary leak, and nutritional reserve.

### Threshold Effect Analysis

Piecewise Cox regression identified distinct threshold effects for both biomarkers (Table 4). For lactate, break points were identified at 5.1 mmol/L for ICU mortality and 5.6 mmol/L for hospital mortality. Below these thresholds, lactate was significantly associated with increased mortality risk (28-day ICU mortality below break point: HR 1.28, 95% CI 1.18-1.38; *P*<.001); above the thresholds, the associations became nonsignificant (above break point: HR 1.04, 95% CI 0.97-1.11; *P*=.24).

In contrast, LAR exhibited more robust and consistent threshold effects. Break points were observed at 1.86 for ICU mortality and 1.67 for hospital mortality. Crucially, the LAR–mortality association remained statistically significant both below (28-day hospital mortality: HR 2.24, 95% CI 1.84-2.74; *P*<.001) and above (HR 1.16, 95% CI 1.06-1.27; *P*<.001) these thresholds, indicating greater prognostic consistency across the full clinical spectrum. Bootstrap resampling (500 iterations, fully adjusted Cox model) confirmed threshold stability: the LAR bootstrap medians were 1.86 (95% CI 1.55-2.15) for ICU mortality and 1.66 (95% CI 1.45-2.05) for hospital mortality, in near-perfect agreement with the primary estimates. Bootstrap threshold stability plots are presented in Multimedia Appendix 1.

**Table 4:** Threshold effect analysis of lactate and LAR^a^ on 28-day ICU^b^ and hospital mortality. Results from multivariable Cox proportional-hazards regression with 28-day administrative censoring. “Linear” = overall linear HR^c^ from Cox regression. “Threshold” refers to piecewise Cox regression with identified break point. Bootstrap analysis (500 resamples) used the same fully adjusted model specification as the primary analysis.

Outcome	Linear	Threshold, HR (95% CI)					Bootstrap (500 resamples)
	Overall HR (95% CI)	Break point (K)	Below K	Above K	Effect Difference	Median K (95% CI)	
Lactate–28-day ICU mortality	1.14 (1.10-1.18)^d^	5.1 mmol/L	1.28 (1.18-1.38)^d^	1.04 (0.97-1.11)	0.81 (0.72-0.92)^d^	5.1 (4.3-5.5) mmol/L	
Lactate–28-day hospital mortality	1.13 (1.10-1.17)^d^	5.6 mmol/L	1.25 (1.17-1.34)^d^	1.03 (0.97-1.10)	0.82 (0.74-0.92)^d^	5.1 (4.5-5.6) mmol/L	
LAR–28-day ICU mortality	1.33 (1.24-1.43)^d^	1.86	2.09 (1.69-2.58)^d^	1.13 (1.02-1.25)^e^	0.54 (0.41-0.71)^d^	1.86 (1.55-2.15)	
LAR–28-day hospital mortality	1.38 (1.29-1.47)^d^	1.67	2.24 (1.84-2.74)^d^	1.16 (1.06-1.27)^d^	0.52 (0.40-0.66)^d^	1.66 (1.45-2.05)	

^a^LAR: lactate-to-albumin ratio.

^b^ICU: intensive care unit.

^c^HR: hazard ratio.

^d^*P*<.001.

^e^*P*=.02.

### Smooth Curve Analysis

Restricted cubic spline analyses (Figures 2 and 3) demonstrated nonlinear relationships for both biomarkers. For lactate, the steepest mortality gradient was observed within the 0-5 mmol/L range, with a plateau and widening CIs above 5 mmol/L, suggesting decreasing predictive reliability at extreme values. For LAR, mortality risk increased steadily across the 0-5 range, plateauing between 5 and 7, with predictive reliability extending to values of 10. These divergent patterns support a complementary clinical role: lactate as an acute-phase monitoring tool (most reliable at concentrations 0-5 mmol/L) and LAR as a sustained prognostic instrument across the full sepsis trajectory.

### Machine Learning–Based 28-Day ICU Mortality Prediction

**Figure 4:**
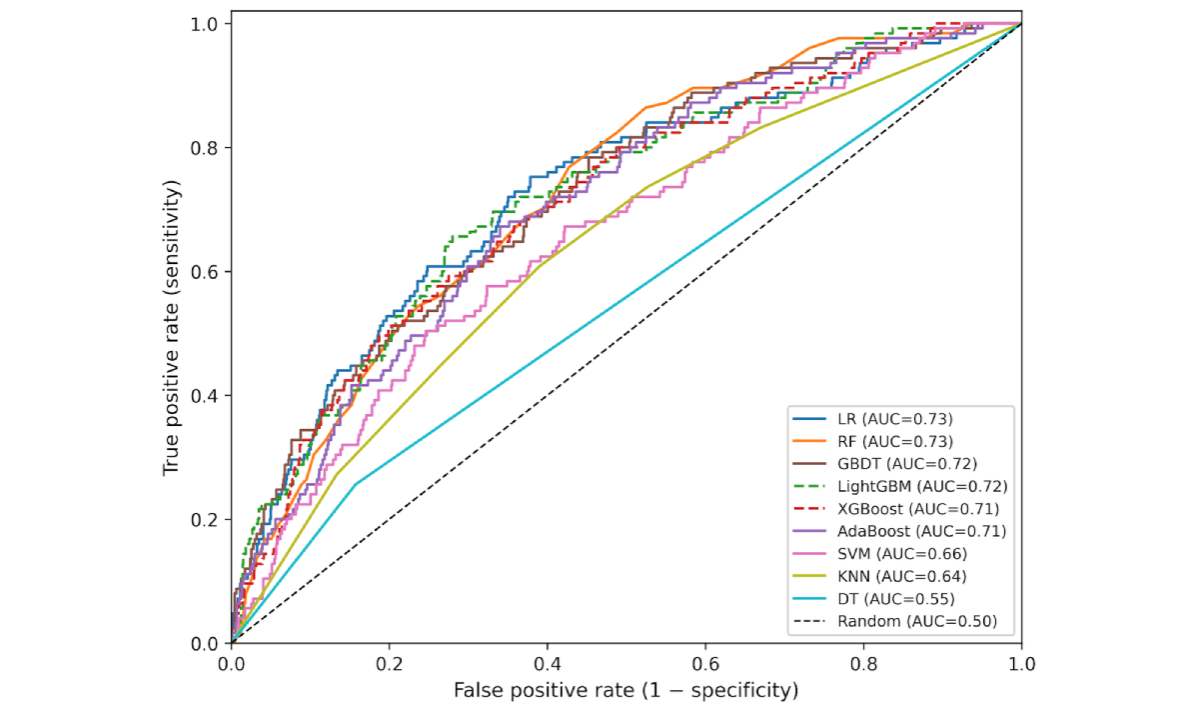
Receiver operating characteristic curves for the 9 machine learning models predicting 28-day intensive care unit mortality among adult patients with sepsis (retrospective cohort study, eICU Collaborative Research Database, n=3637; United States, 2014-2015; held-out test set: n=1092; outcome prevalence: 125/1092, 11.4%). Dashed diagonal line: random classifier (area under the receiver operating characteristic curve [AUC]=0.50). AdaBoost: adaptive boosting; DT: decision tree; GBDT: gradient-boosting decision tree; KNN: k-nearest neighbors; LightGBM: Light Gradient Boosting Machine; LR: logistic regression; RF: random forest; SVM: support vector machine; XGBoost: extreme gradient boosting.

**Figure 5:**
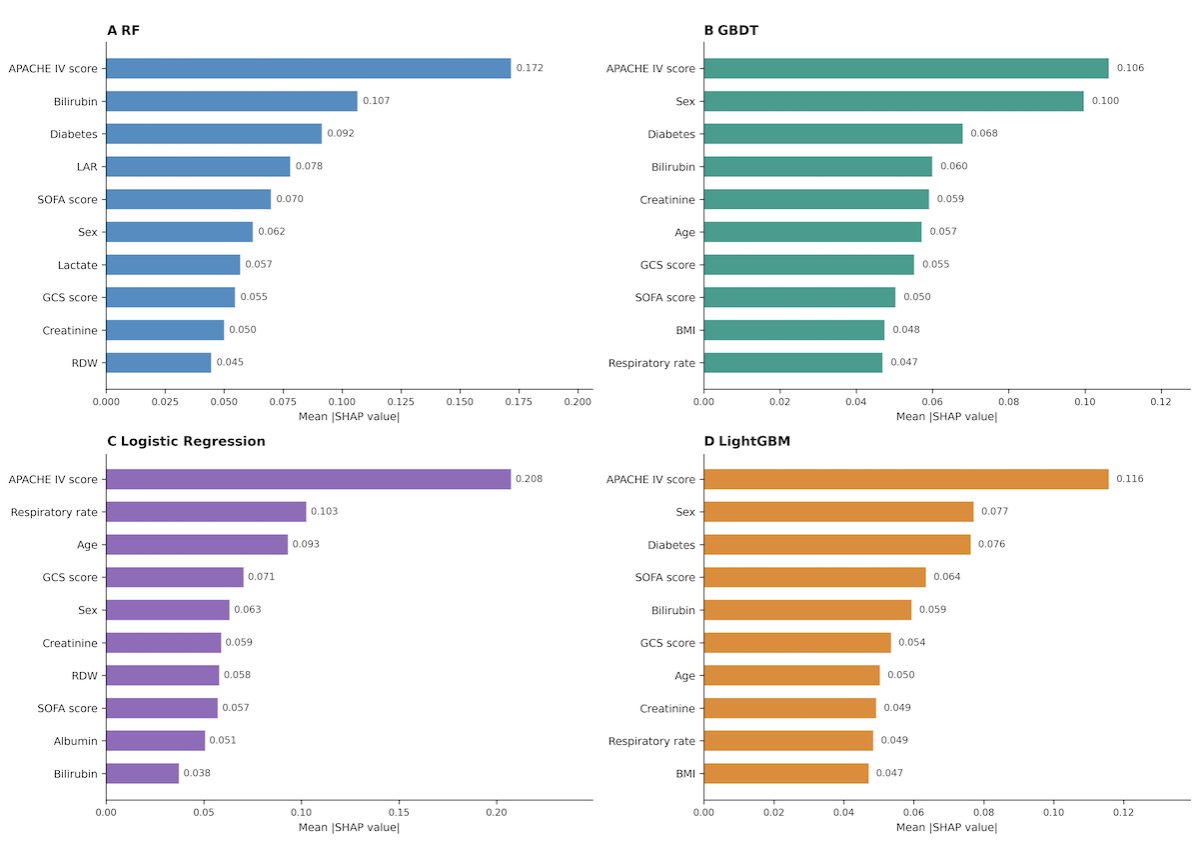
Shapley additive explanations (SHAP) global feature importance for 28-day intensive care unit mortality prediction among adult patients with sepsis (retrospective cohort study, eICU Collaborative Research Database, n=3637; United States, 2014-2015). Top 10 features shown for: (A) random forest (RF), (B) gradient-boosting decision tree (GBDT), (C) logistic regression (LR), and (D) Light Gradient Boosting Machine (LightGBM). Feature importance is expressed as the mean absolute SHAP value. Higher values indicate greater overall contribution to model predictions. Exact values are labeled on each bar. No aggregated residual bar is included. APACHE IV: Acute Physiology and Chronic Health Evaluation IV; GCS: Glasgow Coma Scale; SOFA: Sequential Organ Failure Assessment.

Among 9 machine learning models evaluated on the held-out test set (n=1092; outcome prevalence: 125/1092, 11.4%), LR and RF achieved the joint-highest AUC (0.727 and 0.726, respectively), followed by GBDT (0.725) and LightGBM (0.724). LightGBM demonstrated the best probability calibration with the lowest Brier score (0.096), the only model below the null Brier of 0.101 at the natural test-set prevalence of 11.4% (125/1092); GBDT achieved the highest PR-AUC among all 9 models on the natural held-out test set (0.293). Full performance metrics are presented in Table 5 and ROC curves in Figure 4, calibration curves in Multimedia Appendix 2, and DCA in Multimedia Appendix 3. DCA demonstrated net clinical benefit for the machine learning models over treat-all and treat-none reference strategies across clinically relevant threshold probabilities, supporting their potential utility for individualized risk-stratified decision-making in ICU sepsis management. SHAP analyses across the 4 presented models (Figures 5A-D) consistently identified APACHE IV score as the dominant predictor of 28-day ICU mortality. LAR featured among the top 10 predictive features in the RF model (rank 4), while it fell outside the top 10 in the GBDT, LR, and LightGBM models. This variation across model architectures is consistent with LAR serving as a complementary rather than dominant predictor in the machine learning framework. Beeswarm plots illustrating the direction and magnitude of individual SHAP contributions for each model are provided in Multimedia Appendices 4-7.

**Table 5:** Machine learning model performance—28-day ICU^a^ mortality prediction. F1-score (positive class), sensitivity, and specificity were evaluated at the per-model Youden J threshold (the probability cut-point maximizing sensitivity + specificity − 1) on the held-out test set. AUC^b^, PR-AUC^c^, and Brier score are threshold-free and constitute the primary performance metrics; threshold-dependent metrics are reported for descriptive purposes only. F1: sklearn f1_score(pos_label=1, average='binary').

Model	AUC (95% CI)	PR-AUC (95% CI)	Brier	*F*_1_-score	Sensitivity	Specificity
LR^d^	0.727 (0.679-0.776)	0.290 (0.223-0.370)	0.215	0.321	0.752	0.622
RF^e^	0.726 (0.684-0.772)	0.229 (0.180-0.294)	0.108	0.303	0.768	0.573
GBDT^f^	0.725 (0.681-0.771)	0.293 (0.217-0.375)	0.103	0.297	0.784	0.548
LightGBM^g^	0.724 (0.675-0.776)	0.265 (0.208-0.343)	0.096^h^	0.343	0.656	0.720
XGBoost^i^	0.712 (0.666-0.759)	0.247 (0.189-0.316)	0.103	0.318	0.592	0.725
AdaBoost^j^	0.709 (0.663-0.752)	0.245 (0.186-0.314)	0.218	0.312	0.672	0.660
SVM^k^	0.661 (0.609-0.711)	0.191 (0.147-0.250)	0.128	0.296	0.504	0.754
KNN^l^	0.638 (0.593-0.690)	0.282 (0.225-0.339)	0.292	0.263	0.608	0.610
DT^m^	0.549 (0.508-0.593)	0.258 (0.193-0.319)	0.224	0.207	0.256	0.843

^a^ICU: intensive care unit.

^b^AUC: area under the receiver operating characteristic curve.

^c^PR-AUC: precision-recall AUC.

^d^LR: logistic regression.

^e^RF: random forest.

^f^GBDT: gradient-boosting decision tree.

^g^LightGBM: Light Gradient Boosting Machine.

^h^Only model with Brier score below the null Brier of 0.101 at the natural test-set prevalence of 11.4%.

^i^XGBoost: extreme gradient boosting.

^j^AdaBoost: adaptive boosting.

^k^SVM: support vector machine.

^l^KNN: k-nearest neighbors.

^m^DT: decision tree.

### Machine Learning–Based 28-Day Hospital Mortality Prediction

**Figure 6:**
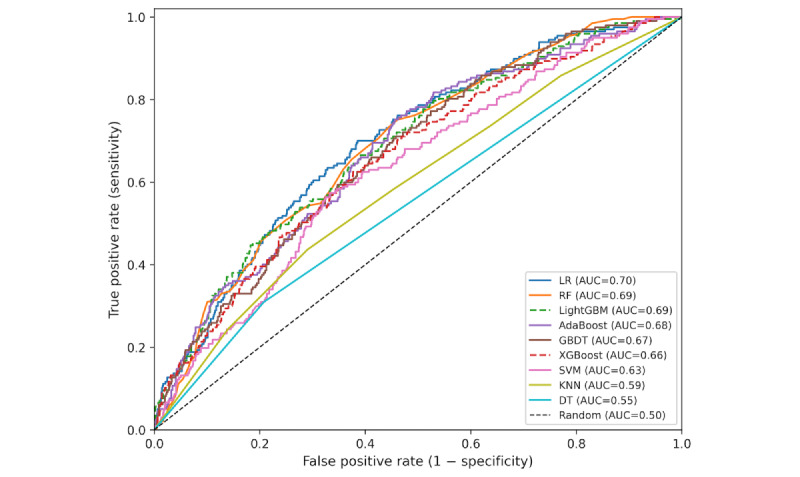
Receiver operating characteristic curves for the 9 machine learning models predicting 28-day hospital mortality among adult patients with sepsis (retrospective cohort study, eICU Collaborative Research Database, n=3637; United States, 2014-2015; held-out test set: n=1092; outcome prevalence: 197/1092, 18%). AdaBoost: adaptive boosting; DT: decision tree; GBDT: gradient-boosting decision tree; KNN: k-nearest neighbors; LightGBM: Light Gradient Boosting Machine; LR: logistic regression; RF: random forest; SVM: support vector machine; XGBoost: extreme gradient boosting.

**Figure 7:**
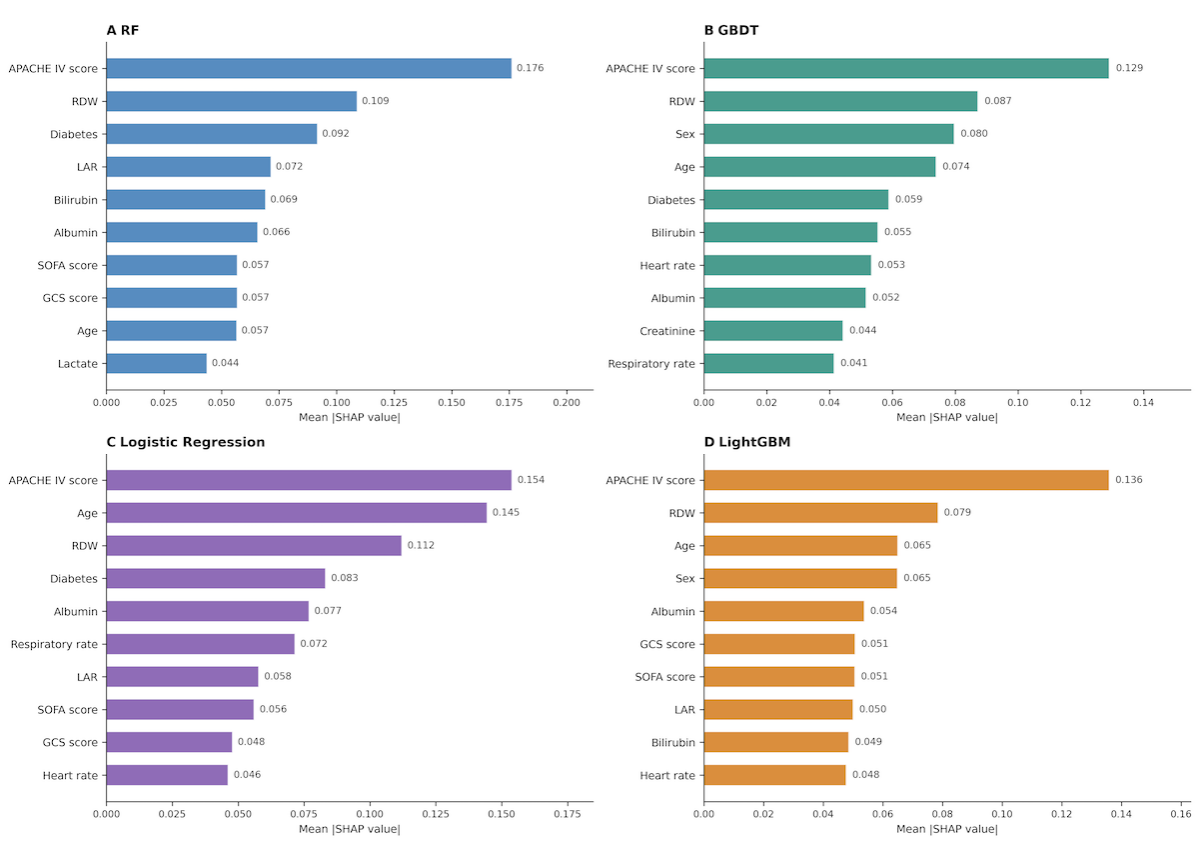
Shapley additive explanations (SHAP) global feature importance for 28-day hospital mortality prediction among adult patients with sepsis (retrospective cohort study, eICU Collaborative Research Database, n=3637; United States, 2014-2015). Top 10 features are shown for: (A) random forest (RF), (B) gradient-boosting decision tree (GBDT), (C) logistic regression (LR), and (D) Light Gradient Boosting Machine (LightGBM). Feature importance is expressed as the mean absolute SHAP value. Higher values indicate greater overall contribution to model predictions. Exact values are labeled on each bar. No aggregated residual bar is included. APACHE IV: Acute Physiology and Chronic Health Evaluation IV; GCS: Glasgow Coma Scale; SOFA: Sequential Organ Failure Assessment.

On the held-out test set for hospital mortality (n=1092; outcome prevalence: 197/1092, 18%), LR achieved the highest AUC (0.699), followed by RF (0.691) and LightGBM (0.688). LightGBM demonstrated the best probability calibration with the lowest Brier score (0.146), the only model below the null Brier of 0.148 at the natural hospital mortality prevalence of 18% (197/1092). Full performance metrics are presented in Table 6 and ROC curves in Figure 6, calibration curves in Multimedia Appendix 8, and DCA in Multimedia Appendix 9. DCA indicated that the machine learning models provided net clinical benefit over treat-all and treat-none reference strategies at threshold probabilities of clinical relevance, suggesting their potential value in identifying patients with sepsis at elevated risk of hospital mortality who may warrant closer monitoring or timely escalation of care. In the SHAP-based feature importance analysis for hospital mortality (Figures 7A-D), APACHE IV score was consistently the leading predictor across all 4 models. LAR was identified in the top 10 features for RF (rank 4), LR (rank 7), and LightGBM (rank 8) but was absent from the GBDT top 10, indicating variable prominence across model architectures consistent with LAR serving as a complementary predictor. Corresponding beeswarm plots for the hospital mortality models are provided in Multimedia Appendices 10-13.

**Table 6:** Machine learning model performance—28-day hospital mortality prediction. F1-score (positive class), sensitivity, and specificity were evaluated at the per-model Youden J threshold (the probability cut-point maximizing sensitivity + specificity − 1) on the held-out test set. AUC^a^, PR-AUC^b^, and Brier score are threshold-free and constitute the primary performance metrics; threshold-dependent metrics are reported for descriptive purposes only. F1: sklearn f1_score(pos_label=1, average='binary'). Performance evaluated on the shared natural held-out test set (30%, n=1092; 28-day hospital mortality prevalence 197/1092, 18%; null Brier=0.148).

Model	AUC (95% CI)	PR-AUC (95% CI)	Brier	*F*_1_-score	Sensitivity	Specificity
LR^c^	0.699 (0.660-0.739)	0.334 (0.275-0.402)	0.220	0.406	0.701	0.615
RF^d^	0.691 (0.651-0.731)	0.294 (0.248-0.349)	0.152	0.391	0.751	0.539
LightGBM^e^	0.688 (0.649-0.727)	0.344 (0.288-0.405)	0.146^f^	0.386	0.665	0.609
AdaBoost^g^	0.680 (0.639-0.722)	0.315 (0.260-0.380)	0.226	0.393	0.751	0.544
GBDT^h^	0.673 (0.634-0.713)	0.309 (0.256-0.372)	0.151	0.375	0.711	0.542
XGBoost^i^	0.660 (0.617-0.702)	0.303 (0.246-0.364)	0.159	0.374	0.624	0.623
SVM^j^	0.632 (0.587-0.672)	0.263 (0.219-0.320)	0.191	0.372	0.574	0.667
KNN^k^	0.592 (0.550-0.640)	0.324 (0.277-0.372)	0.326	0.317	0.437	0.709
DT^l^	0.551 (0.515-0.588)	0.341 (0.287-0.393)	0.295	0.275	0.310	0.792

^a^AUC: area under the receiver operating characteristic curve.

^b^PR-AUC: precision-recall AUC.

^c^LR: logistic regression.

^d^RF: random forest.

^e^LightGBM: Light Gradient Boosting Machine.

^f^Only model with Brier score below the null Brier of 0.148 at the natural test-set prevalence of 18% (197/1092).

^g^AdaBoost: adaptive boosting.

^h^GBDT: gradient-boosting decision tree.

^i^XGBoost: extreme gradient boosting.

^j^SVM: support vector machine.

^k^KNN: k-nearest neighbors.

^l^DT: decision tree.

### Ancillary Analyses

#### Collinearity

VIFs were computed to characterize the degree of collinearity among predictors. VIF values for lactate and LAR were 5.09. Although this falls below the conventional threshold of 10, LAR is algebraically derived from lactate and albumin (LAR = lactate ÷ albumin); consequently, simultaneous unpenalized inclusion of all 3 variables in a single LR model would preclude valid coefficient identification regardless of the VIF magnitude. This is why, in Table 3, LAR, lactate, and albumin were entered as exposures in 3 separate models within each lactate stratum, and why L2 regularization was applied to the machine learning LR model, which jointly shrinks collinear coefficient estimates and yields stable predictions despite their algebraic dependence.

#### E-Value Analysis

For the LAR–hospital mortality association (OR 1.43, 95% CI 1.29-1.59), an unmeasured confounder would require associations of risk ratio ≥2.21 with both LAR and mortality to fully explain the observed effect.

### External Validation and Generalizability

**Figure 8:**
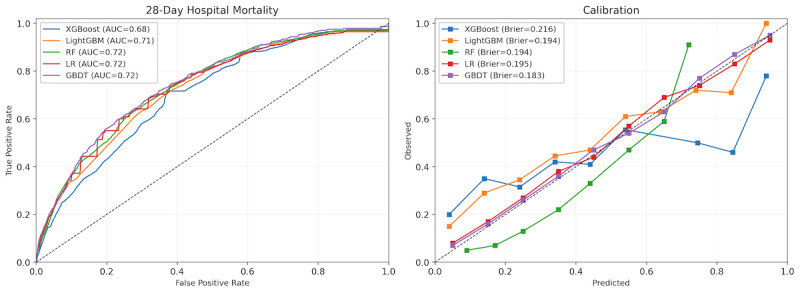
External validation on Medical Information Mart for Intensive Care IV (MIMIC-IV): receiver operating characteristic and calibration for 28-day hospital mortality. Trained on eICU Collaborative Research Database (n=3637), tested on MIMIC-IV (n=3300). GBDT: gradient-boosting decision tree; LightGBM: Light Gradient Boosting Machine; LR: logistic regression; RF: random forest; XGBoost: extreme gradient boosting.

**Figure 9:**
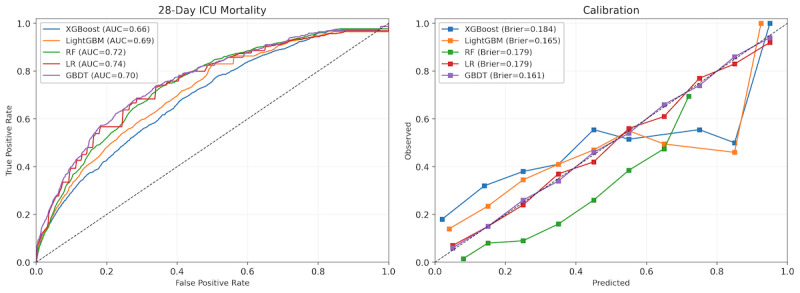
External validation on Medical Information Mart for Intensive Care IV (MIMIC-IV): receiver operating characteristic and calibration for 28-day intensive care unit (ICU) mortality. Logistic regression (LR) achieved the highest external area under the receiver operating characteristic curve (AUC) of 0.74. GBDT: gradient-boosting decision tree; LightGBM: Light Gradient Boosting Machine; LR: logistic regression; RF: random forest; XGBoost: extreme gradient boosting.

Models trained on the eICU cohort (n=3637) demonstrated robust performance when applied to the independent MIMIC-IV cohort (n=3300; Beth Israel Deaconess Medical Center, Boston; 2008-2019), despite differences in calendar period, institutional setting, and case-mix. The MIMIC-IV cohort had a mean age of 67.3 (SD 15.7) years, 45% (1485/3300) female, with 28-day ICU mortality of 21.9% and hospital mortality of 29.5%. For 28-day hospital mortality, LR and RF achieved the highest external AUC of 0.723, followed by GBDT (0.716) and LightGBM (0.711), with Brier scores of 0.183-0.195. For 28-day ICU mortality, LR achieved AUC 0.739, followed by RF (0.725) and GBDT (0.698). These external validation AUCs were comparable to or slightly higher than internal validation (0.70-0.74), confirming robust generalizability across independent multicenter datasets. Calibration curves demonstrated reasonable agreement between predicted and observed probabilities (Figures 8 and 9). Future prospective studies should evaluate whether LAR-guided early intervention improves outcomes. Integration of dynamic LAR trajectories may further enhance predictive accuracy.

## Discussion

### Overview

This study provides a methodologically rigorous evaluation of lactate and the LAR as prognostic biomarkers in ICU patients with sepsis. Our findings demonstrate that LAR has statistically significantly higher stand-alone discriminative performance than lactate, as formally confirmed by the DeLong test for both 28-day hospital mortality (*P*<.001) and ICU mortality (*P*<.001). Although the absolute differences in AUC are modest (ΔAUC=0.029 and 0.021), which is expected given that LAR is algebraically derived from lactate, their consistent statistical robustness across both outcome definitions in a cohort of 3637 patients provides strong evidence to support the preferential use of LAR over lactate alone as a single biomarker for sepsis risk stratification. Of note, a statistically nominally significant interaction was observed between APACHE IV severity tertile and the LAR in predicting hospital mortality (*P*=.02). However, given that this interaction was identified as part of a hypothesis-generating exploratory subgroup analysis involving multiple simultaneous tests across 2 outcome end points, this finding should be interpreted with caution. It does not survive correction for multiple comparisons and may represent a false-positive discovery. This observation is presented solely as an exploratory hypothesis to be evaluated in dedicated prospective studies and should not be used to guide clinical decision-making without independent replication.

Some retrospective observational studies have confirmed the association between elevated LAR and increased mortality in sepsis [23], while Liu et al [24] established lactate as a crucial predictor of mortality. Building on this foundation, this study extends existing evidence by providing the first formal paired AUC comparison demonstrating that the observed numerical advantage of LAR over lactate achieves statistical significance. In addition, threshold effect analysis reveals that LAR is particularly valuable in patients with lactate levels <4 mmol/L, where albumin contributes independent prognostic information not captured by lactate alone. This finding is consistent with observations reported by Bou Chebl et al [25,26]. Furthermore, machine learning models incorporating LAR consistently outperformed traditional scoring systems when used as stand-alone predictors, and SHAP-based interpretability supports their potential clinical adoption [27].

The biological plausibility of these findings is well supported. Lactate reflects the acute metabolic consequences of tissue hypoperfusion and is most discriminative within the 0-5 mmol/L range [28]. In contrast, albumin, as a negative acute-phase protein, declines progressively during sepsis due to systemic inflammation, capillary leak, and altered synthesis [29,30]. The combination of elevated lactate and decreased albumin, therefore, captures both acute metabolic derangement and subacute inflammatory burden, providing a more comprehensive representation of sepsis pathophysiology. This likely explains why LAR demonstrates statistically significantly higher, albeit modest, predictive performance compared to lactate alone, particularly below critical lactate thresholds where lactate may not yet reflect severe physiological disturbance [31,32].

Several limitations should be acknowledged. First, the eICU-CRD database spans 2014-2015; however, the core pathophysiological principles underlying lactate and albumin remain applicable. Second, the retrospective design precludes causal inference. Third, the exclusion of patients with ICU stays <48 hours may introduce survivorship bias. Fourth, biomarker values were based on measurements obtained within 24 hours of ICU admission; a sensitivity analysis excluding 142 patients (3.9%) whose first measurement preceded ICU admission by >6 hours confirmed no material effect of measurement-timing misclassification (ΔAUC<0.01). Fifth, bootstrap resampling (500 iterations, same fully adjusted Cox model specification as the primary analysis) confirmed threshold stability: median LAR thresholds of 1.86 (95% CI 1.55-2.15) for ICU mortality and 1.66 (95% CI 1.45-2.05) for hospital mortality, in close agreement with the primary piecewise Cox estimates (1.86 and 1.67, respectively), confirming that the identified break points are not artefacts of the point-estimate procedure. Sixth, while SHAP-based interpretability mitigates some of the complexity inherent in machine learning models, barriers to clinical implementation remain. Seventh, probability calibration varied substantially across models for 28-day ICU mortality: LightGBM demonstrated the best probability calibration (Brier score 0.096) and was the only model to fall below the null Brier of 0.101 for ICU mortality (and 0.148 for hospital mortality). The remaining 8 models exceeded the null Brier in both outcomes, indicating that their predicted probabilities are less reliable than their AUC values suggest. The unusually high LR Brier score, which exceeds twice the null Brier value of 0.101, warrants specific explanation: SMOTE oversampling applied during training artificially equalizes class proportions, shifting the model’s baseline predicted probability upward; without post hoc recalibration, this intercept shift persists when the model is applied to the naturally imbalanced test set (prevalence: 125/1092, 11.4%), producing systematically inflated predicted probabilities and a correspondingly elevated Brier score. This is a procedural artefact of SMOTE without recalibration rather than an intrinsic limitation of LR on tabular data of this size. The same mechanism applies to the LR model for 28-day hospital mortality, where the Brier score of 0.220 similarly exceeds the null Brier value of approximately 0.148 at the natural prevalence of 18% (197/1092); the SMOTE-induced intercept shift is an equally applicable explanation in that setting, and the same recommendation for post hoc recalibration before clinical deployment applies to both outcome models. Platt scaling or isotonic regression applied to a held-back calibration partition is recommended before any clinical deployment of these models. Eighth, the training cohort (eICU-CRD) has a hospital mortality rate of 18.3% (666/3637), whereas the MIMIC-IV external validation cohort has a substantially higher rate of 29.5% (972/3300). This prevalence shift introduces systematic intercept miscalibration: models trained on the lower-prevalence eICU data tend to underpredict absolute mortality risk in the higher-acuity MIMIC-IV population, as reflected in the external validation calibration plots. Recalibration methods—such as logistic recalibration or Bayesian intercept updating—are recommended before deploying these models in populations with substantially different baseline risk profiles. Ninth, the nominally significant APACHE IV × LAR interaction for hospital mortality (*P* for interaction=.02) was identified as part of a hypothesis-generating exploratory subgroup analysis involving multiple simultaneous tests across 2 outcome end points; it does not survive correction for multiple comparisons and should not be considered a confirmed subgroup finding without independent prospective replication. A further methodological consideration concerns the subgroup analysis restricted to patients with lactate <2 mmol/L (Table 3), in which lactate loses statistical significance while LAR remains highly predictive. This pattern likely reflects a range restriction artifact: conditioning on lactate values below a threshold artificially truncates lactate variance, attenuating its estimated regression coefficient. LAR retains variability within this subgroup due to albumin fluctuations, preserving its predictive signal. The apparent superiority of LAR in this subgroup should therefore be interpreted as a mathematical consequence of the restricted sampling frame, rather than evidence of intrinsic superiority over lactate. Tenth, the external validation is subject to an important methodological constraint: APACHE IV—the single most influential predictor across all eICU-trained models per SHAP analyses—is not natively available in MIMIC-IV. Its absence was handled via KNN imputation using statistics from the eICU-CRD training partition; specifically, because APACHE IV was structurally absent for all MIMIC-IV patients (an entirely missing column rather than random missingness), the KNN Imputer assigned each patient the mean APACHE IV of its k=5 nearest eICU training-set neighbors identified on the 17 remaining shared features, producing near-constant imputed values due to the distributional overlap between the 2 ICU sepsis cohorts, and thereby substantially reducing the discriminative contribution of APACHE IV in the external validation setting. The external validation AUCs (ICU mortality: 0.663-0.739; hospital mortality: 0.676-0.723), therefore, reflect predominantly the predictive capacity of the remaining 17 shared clinical features—vital signs, laboratory indices, SOFA score, and comorbidity indicators—rather than the full eICU feature set. Future external validation should prioritize cohorts with native APACHE IV recording or prospectively evaluate model adaptation using equivalent severity scores. Eleventh, although external validation using MIMIC-IV supports broader applicability, generalizability beyond US institutions requires further prospective validation. Twelfth, it should be noted that the stand-alone DeLong AUC comparison and the descriptive ORs and HRs reported across differently scaled exposures represent unadjusted comparisons between biomarkers; the appropriately controlled inferences in this study derive from the multivariable regression and machine learning analyses.

These findings have important clinical implications. LAR can be readily calculated from 2 routinely available laboratory parameters obtained at ICU admission, without additional cost or procedural burden. The demonstrated superiority of LAR over lactate suggests that it may serve as a more informative single biomarker for early risk stratification. Because the APACHE IV × LAR interaction (*P* for interaction=.02) reflects within-biomarker effect modification rather than formal between-biomarker superiority, and should be interpreted with caution given the multiple-testing context, it raises the exploratory hypothesis that LAR may add particular value in patients who do not initially appear critically ill, particularly those with moderately elevated lactate (<4 mmol/L) in whom hypoalbuminemia may indicate an underlying inflammatory burden not reflected by lactate alone. If confirmed by prospective studies, this would suggest that routine calculation of LAR at ICU admission could facilitate earlier recognition of high-risk patients and prompt more timely clinical intervention. Moreover, the identified LAR threshold of 1.67 for hospital mortality provides a clinically actionable cutoff. Patients with LAR>1.67 exhibited significantly higher 28-day mortality across strata, in contrast to lactate, whose prognostic utility diminished above 5.6 mmol/L. Accordingly, LAR may serve as a complementary risk stratification tool alongside lactate monitoring, particularly when lactate levels are within the normal or mildly elevated range. Future prospective studies are warranted to determine whether LAR-guided management strategies can improve clinical outcomes.

### Conclusions

This large-scale retrospective study of 3637 adult ICU patients with sepsis provides formally validated evidence that LAR, a mixed-unit composite index (mmol/L per g/dL) integrating acute metabolic derangement with hypoalbuminemia, demonstrates statistically significantly higher discrimination than lactate alone for 28-day sepsis mortality. DeLong test confirmed this advantage for both 28-day hospital mortality (*P*<.001; ΔAUC=0.029) and 28-day ICU mortality (*P*<.001; ΔAUC=0.021). While the absolute AUC improvement is modest, its consistent statistical significance across both outcomes in a large multicenter cohort provides formal justification to recommend LAR over lactate as the preferred single prognostic biomarker for sepsis risk stratification.

Lactate remains a reliable acute-phase monitoring tool within the 0-5 mmol/L range, while LAR offers more consistent prognostic information across the full spectrum of critical illness severity. The nominally significant APACHE IV × LAR interaction (*P*=.02) remains an exploratory hypothesis requiring prospective validation, as detailed in the discussion. The identified LAR threshold of 1.67 for hospital mortality provides a clinically actionable risk-stratification cut point that retains prognostic significance both below and above the threshold, unlike lactate, which loses significance above 5.6 mmol/L.

Nine interpretable machine learning models identified LAR among the top 10 predictive features in 3 of 4 models for hospital mortality and 1 of 4 models for ICU mortality, consistent with LAR functioning as a complementary rather than dominant predictor within the machine learning framework. External validation on MIMIC-IV confirmed generalizability across independent institutions and patient populations. These findings support integrating LAR calculation into routine ICU admission protocols, particularly for hemodynamically stable patients with mildly elevated lactate, where early, accurate risk stratification is most clinically impactful. Future prospective studies should evaluate whether LAR-guided clinical decision support improves patient outcomes in real-world critical care settings.

## Appendix

Multimedia Appendix 1 Bootstrap threshold stability plots (500 resamples, fully adjusted Cox model)—lactate and lactate-to-albumin ratio.

Multimedia Appendix 2 Calibration curves—28-day intensive care unit mortality prediction.

Multimedia Appendix 3 Decision curve analysis—28-day intensive care unit mortality prediction.

Multimedia Appendix 4 Shapley additive explanations (SHAP) beeswarm plot (random forest)—28-day intensive care unit mortality prediction.

Multimedia Appendix 5 Shapley additive explanations (SHAP) beeswarm plots (gradient-boosting decision tree)—28-day intensive care unit mortality prediction.

Multimedia Appendix 6 Shapley additive explanations (SHAP) beeswarm plot (logistic regression)—28-day intensive care unit mortality prediction.

Multimedia Appendix 7 Shapley additive explanations (SHAP) beeswarm plot (Light Gradient Boosting Machine [LightGBM])—28-day intensive care unit mortality prediction.

Multimedia Appendix 8 Calibration curves: curve analysis—28-day hospital mortality prediction.

Multimedia Appendix 9 Decision curve analysis—28-day hospital mortality prediction.

Multimedia Appendix 10 Shapley additive explanations (SHAP) beeswarm plot (random forest)—28-day hospital mortality prediction.

Multimedia Appendix 11 Shapley additive explanations (SHAP) beeswarm plot (gradient-boosting decision tree)—28-day hospital mortality prediction.

Multimedia Appendix 12 Shapley additive explanations (SHAP) beeswarm plot (logistic regression)—28-day hospital mortality prediction.

Multimedia Appendix 13 Shapley additive explanations (SHAP) beeswarm plot (Light Gradient Boosting Machine [LightGBM])—28-day hospital mortality prediction.

## Abbreviations

AdaBoost: adaptive boosting
APACHE IV: Acute Physiology and Chronic Health Evaluation IV
AUC: area under the receiver operating characteristic curve
DCA: decision curve analysis
eICU-CRD: eICU Collaborative Research Database
GBDT: gradient-boosting decision tree
GCS: Glasgow Coma Scale
HIPAA: Health Insurance Portability and Accountability Act
HR: hazard ratio
ICD-9: International Classification of Diseases, Ninth Revision
ICU: intensive care unit
KNN: k-nearest neighbors
LAR: lactate-to-albumin ratio
LightGBM: Light Gradient Boosting Machine
LR: logistic regression
MAP: mean arterial pressure
MIMIC-IV: Medical Information Mart for Intensive Care IV
OR: odds ratio
PR-AUC: precision-recall AUC
RDW: red cell distribution width
RF: random forest
ROC: receiver operating characteristic
SHAP: Shapley additive explanations
SMOTE: Synthetic Minority Oversampling Technique
SOFA: Sequential Organ Failure Assessment
STROBE: Strengthening the Reporting of Observational Studies in Epidemiology
SVM: support vector machine
VIF: variance inflation factors
WBC: white blood cell
XGBoost: extreme gradient boosting

## Data Availability

The datasets analyzed during this study are available in the PhysioNet repository: eICU Collaborative Research Database (eICU-CRD; [33]) and Medical Information Mart for Intensive Care IV (MIMIC-IV; [34]).
