# Supplementary figures and images for: Machine Learning–Augmented Traditional Analysis of Lactate vs Lactate-to-Albumin Ratio for Predicting Mortality Risk in Patients With Sepsis: Large-Scale Retrospective Study
Source: JMIR Med Inform. 2026 Jul 16;14:e82230. doi: 10.2196/82230 (PMC13424758; doi:10.2196/82230)

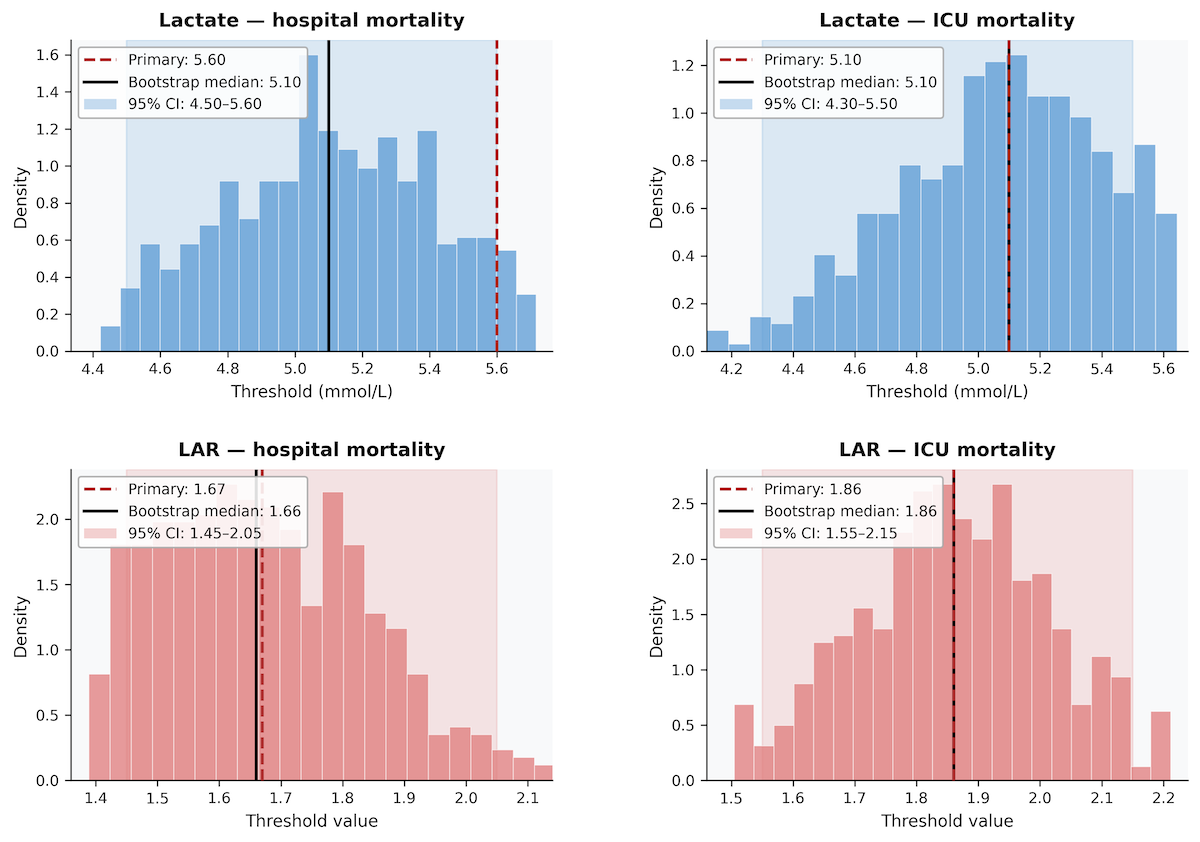

Supplement: Multimedia Appendix 1 [file medinform_v14i1e82230_app1.png]

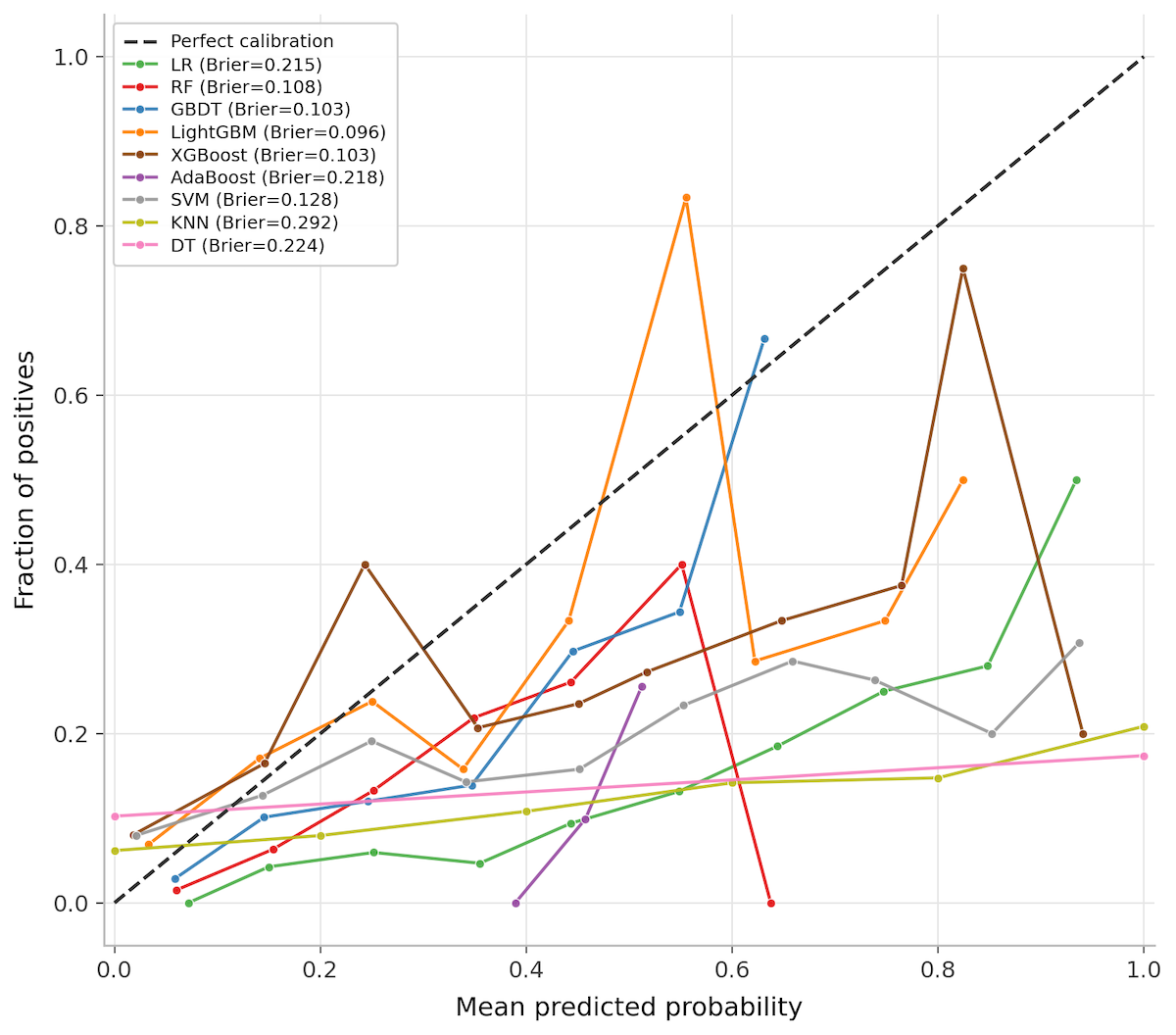

Supplement: Multimedia Appendix 2 [file medinform_v14i1e82230_app2.png]

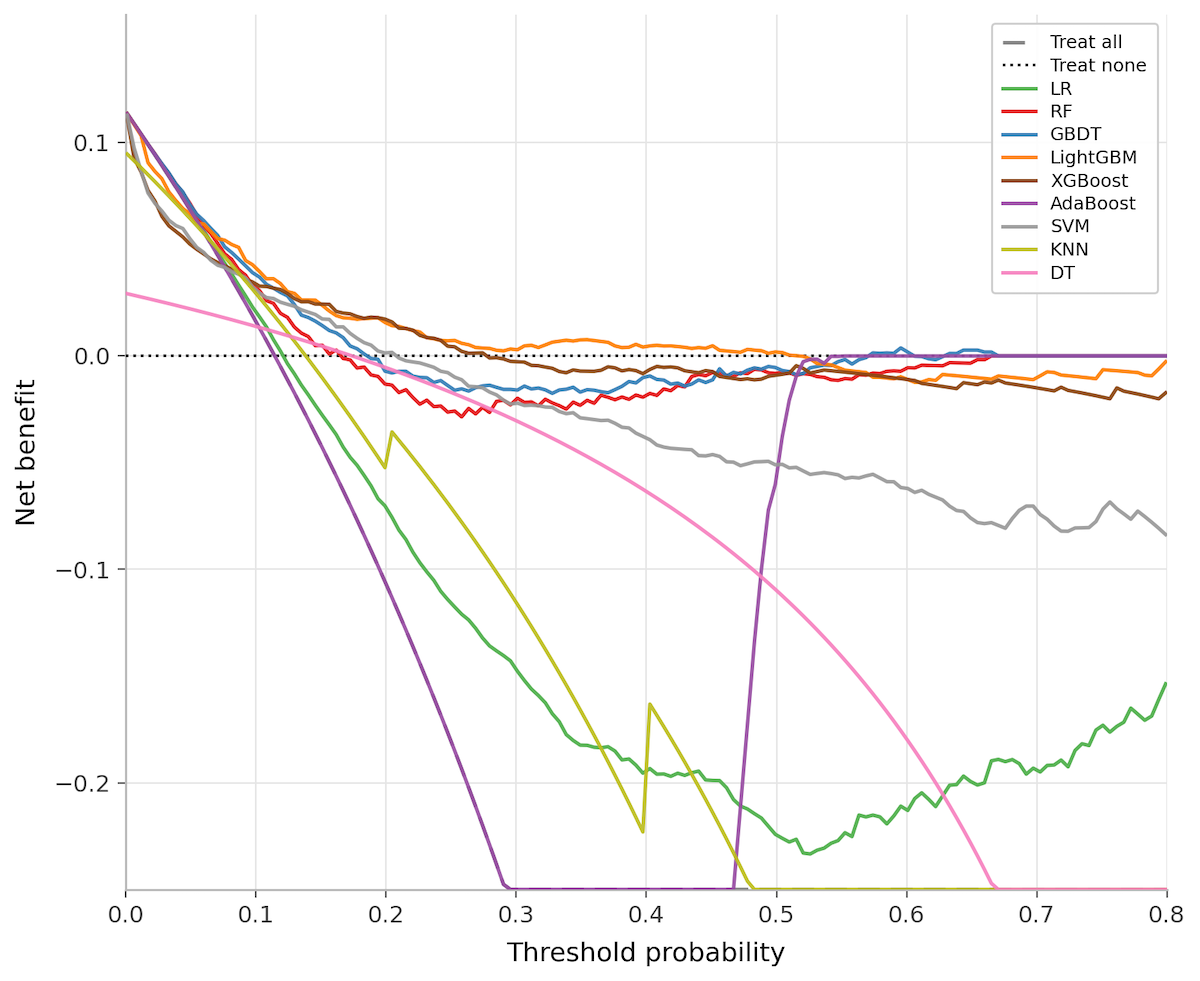

Supplement: Multimedia Appendix 3 [file medinform_v14i1e82230_app3.png]

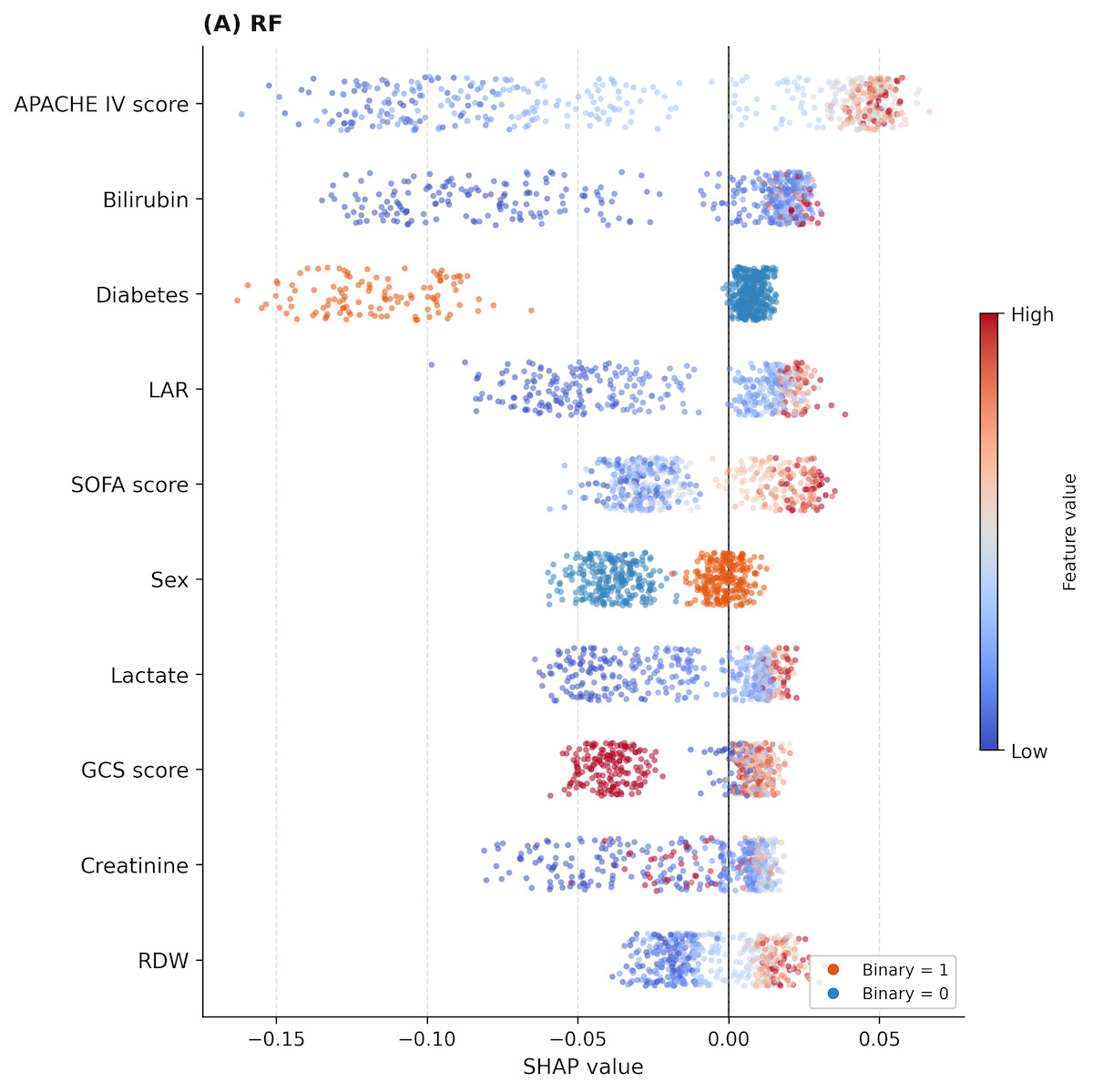

Supplement: Multimedia Appendix 4 [file medinform_v14i1e82230_app4.png]

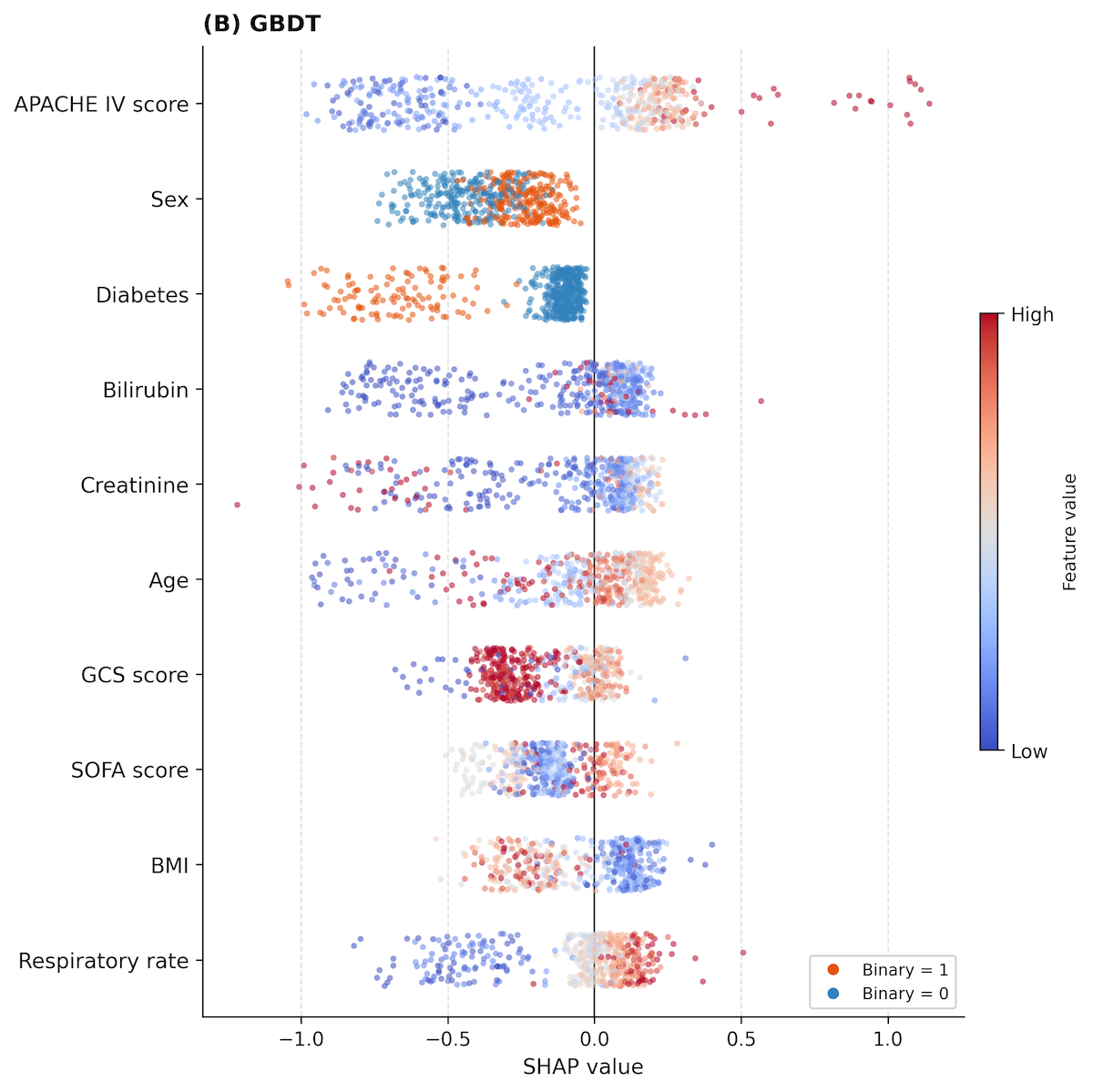

Supplement: Multimedia Appendix 5 [file medinform_v14i1e82230_app5.png]

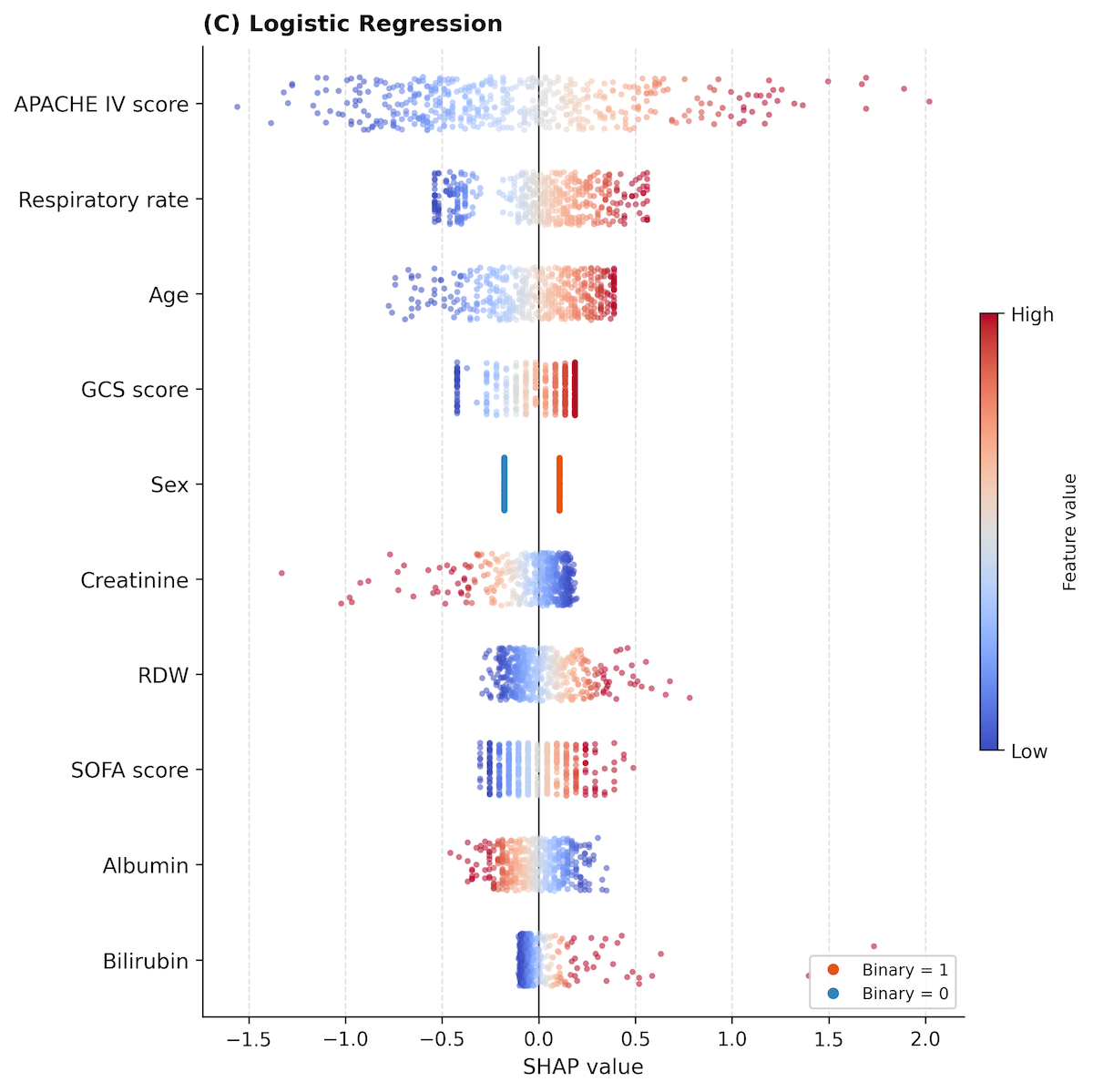

Supplement: Multimedia Appendix 6 [file medinform_v14i1e82230_app6.png]

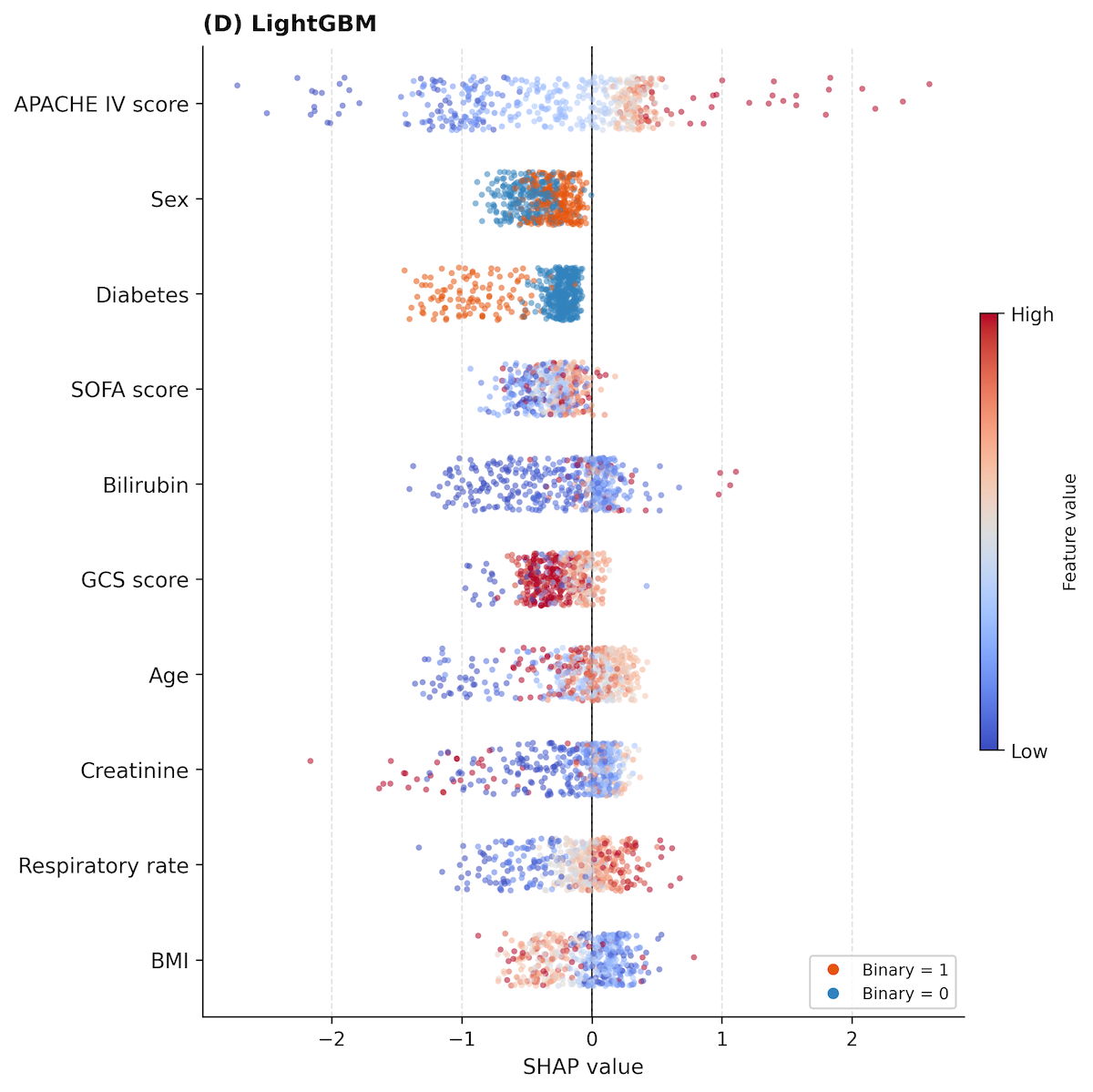

Supplement: Multimedia Appendix 7 [file medinform_v14i1e82230_app7.png]

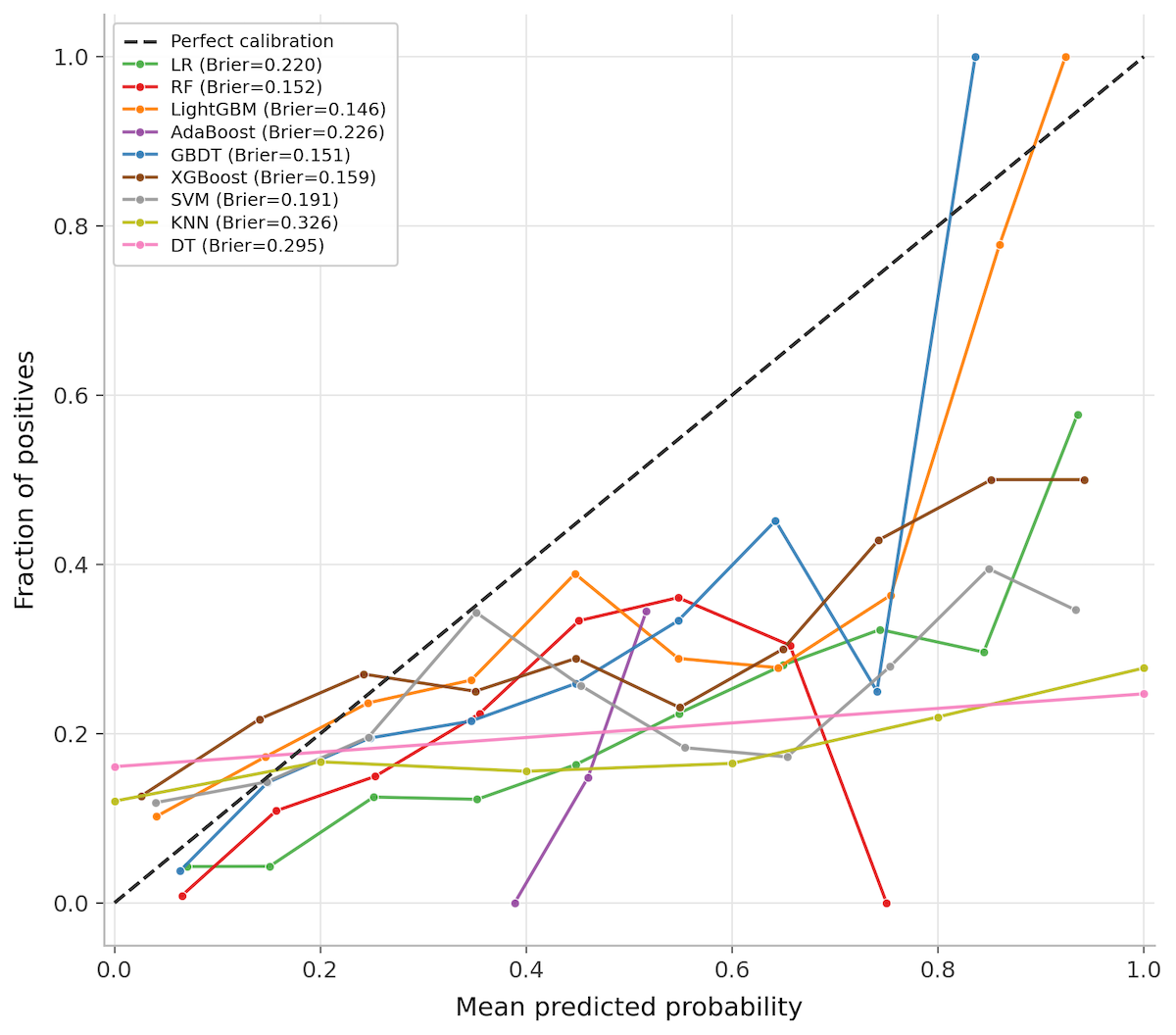

Supplement: Multimedia Appendix 8 [file medinform_v14i1e82230_app8.png]

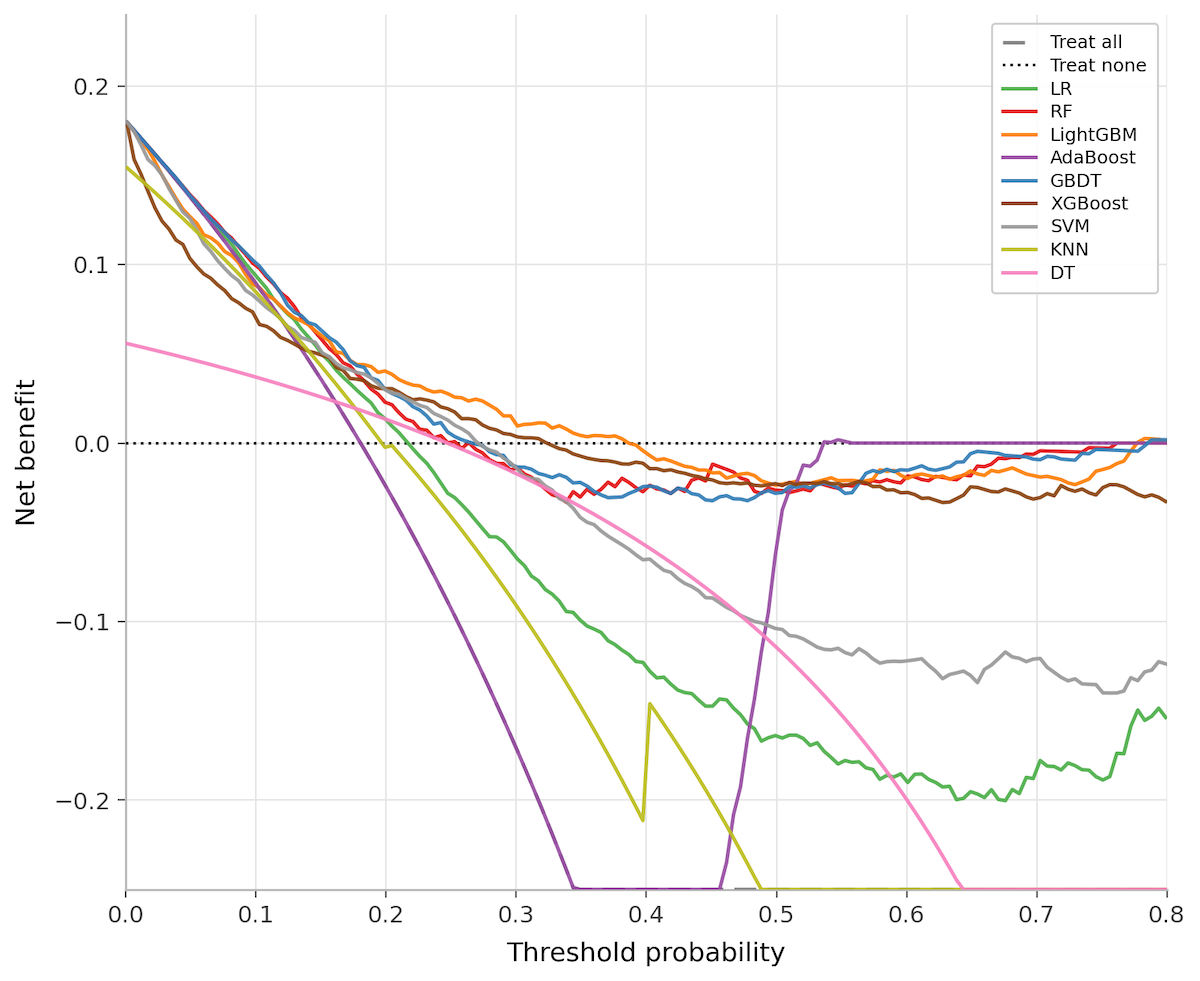

Supplement: Multimedia Appendix 9 [file medinform_v14i1e82230_app9.png]

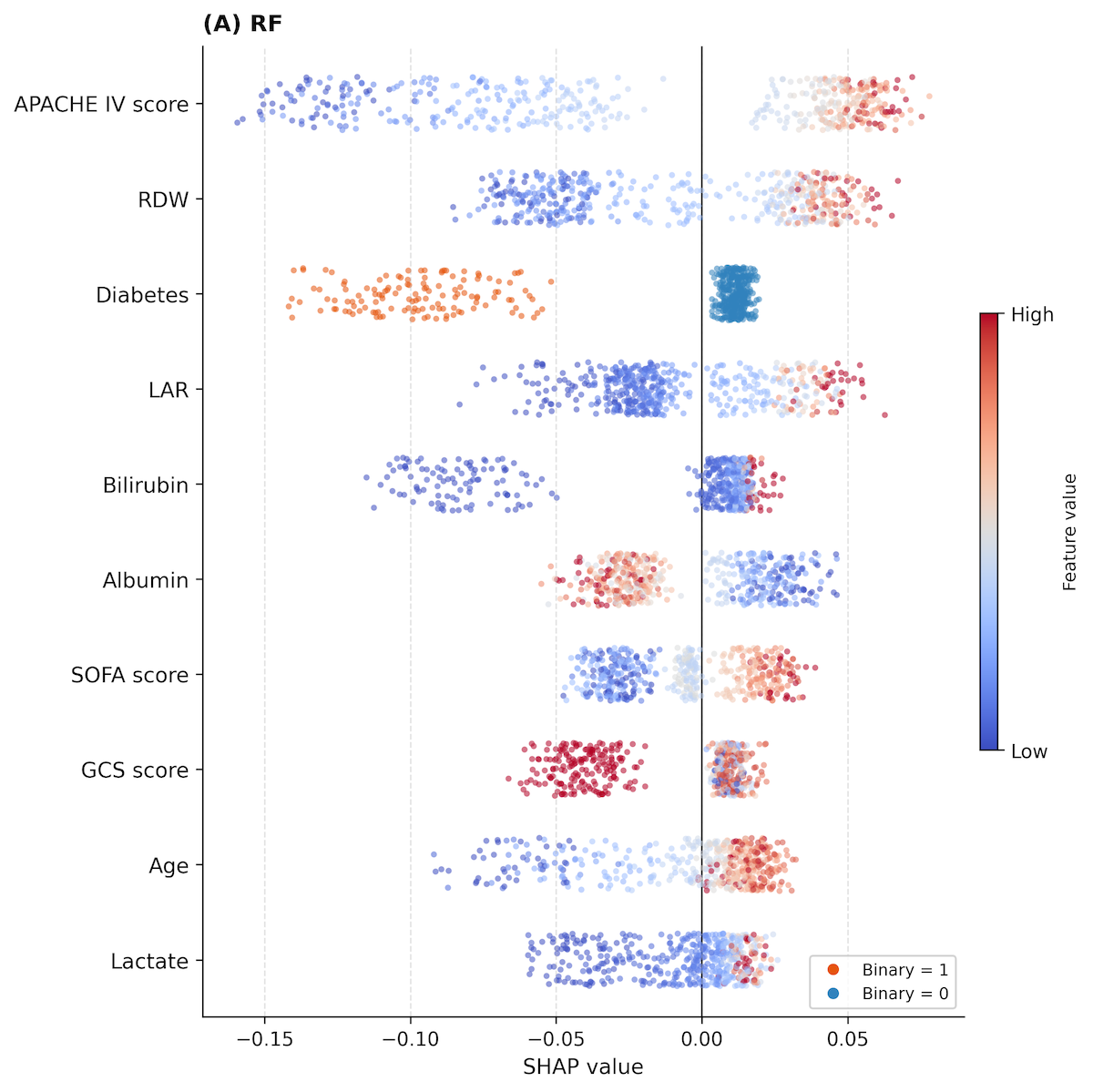

Supplement: Multimedia Appendix 10 [file medinform_v14i1e82230_app10.png]

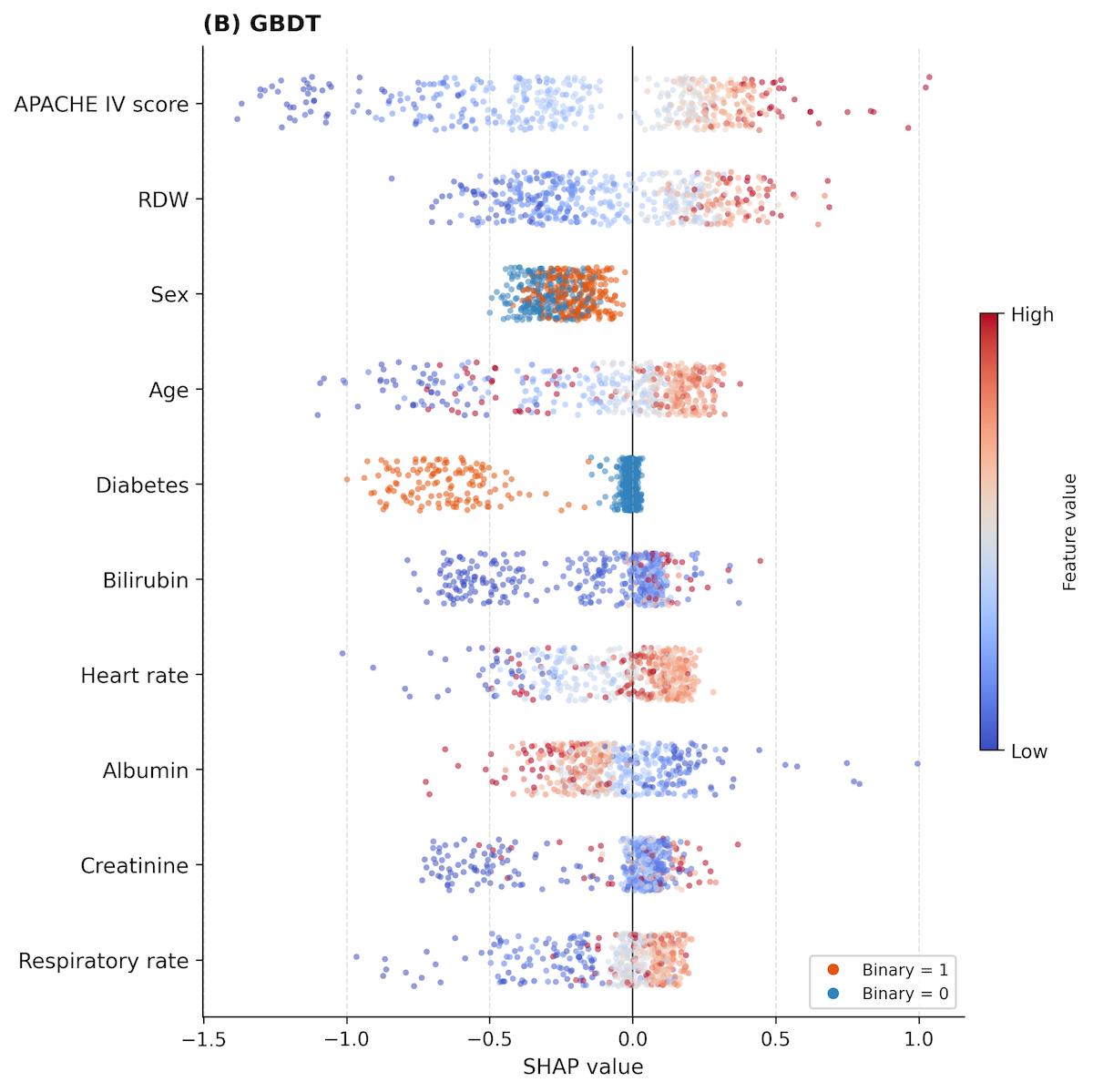

Supplement: Multimedia Appendix 11 [file medinform_v14i1e82230_app11.png]

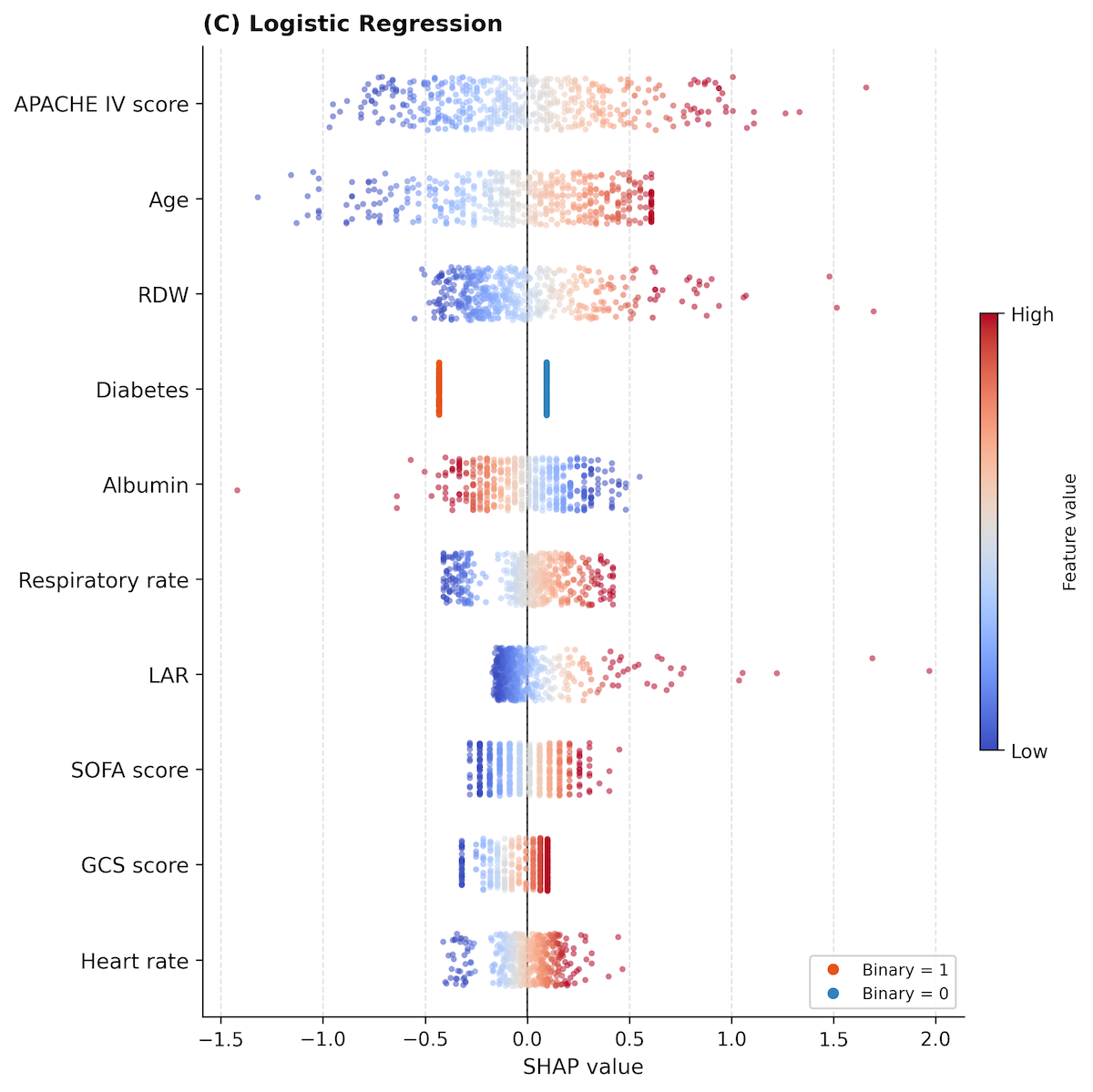

Supplement: Multimedia Appendix 12 [file medinform_v14i1e82230_app12.png]

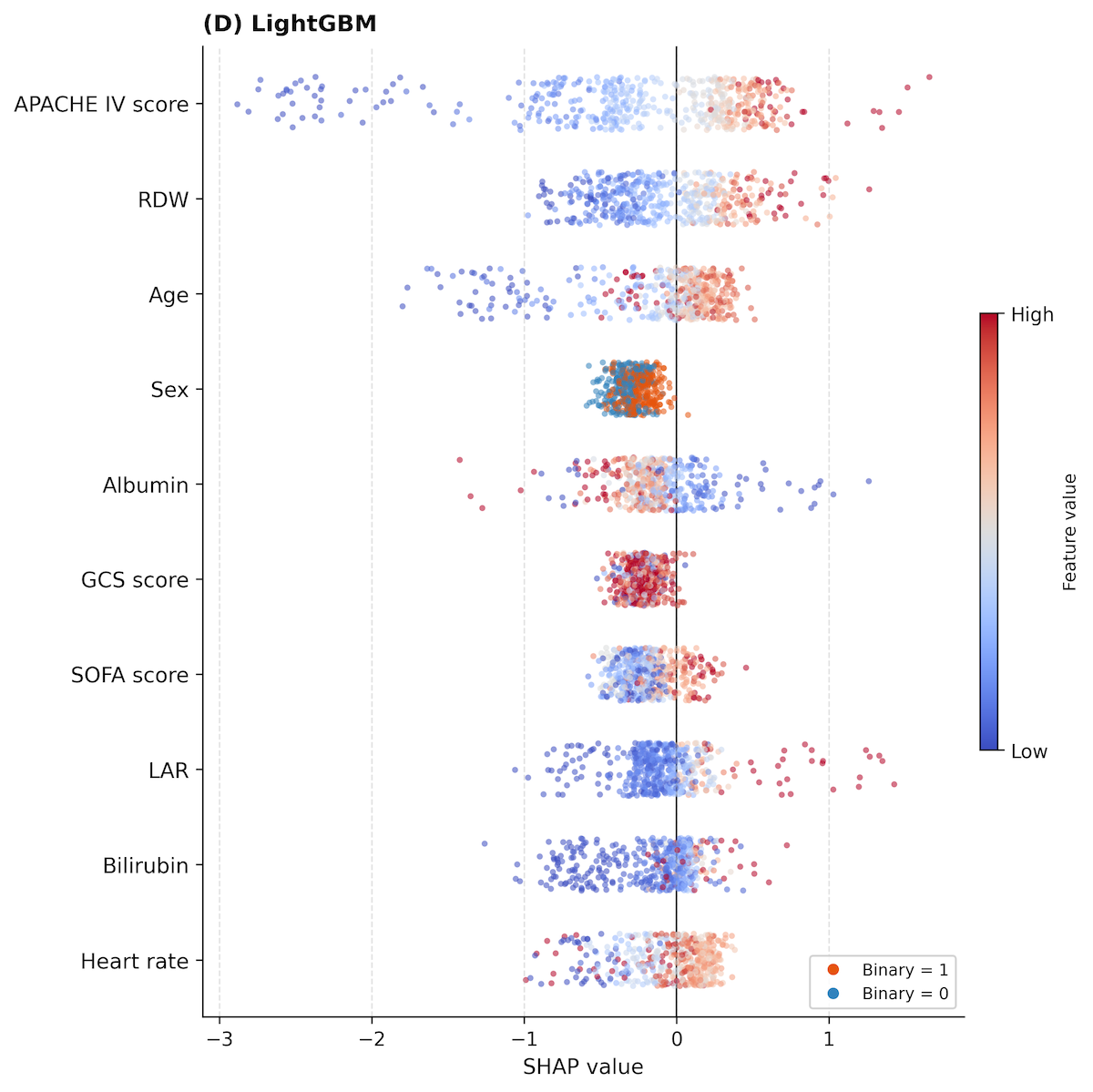

Supplement: Multimedia Appendix 13 [file medinform_v14i1e82230_app13.png]
